# Clinical and genetic architecture of a large cohort with auditory neuropathy

**DOI:** 10.1007/s00439-024-02652-7

**Published:** 2024-03-08

**Authors:** Hongyang Wang, Liping Guan, Xiaonan Wu, Jing Guan, Jin Li, Nan Li, Kaili Wu, Ya Gao, Dan Bing, Jianguo Zhang, Lan Lan, Tao Shi, Danyang Li, Wenjia Wang, Linyi Xie, Fen Xiong, Wei Shi, Lijian Zhao, Dayong Wang, Ye Yin, Qiuju Wang

**Affiliations:** 1 Senior Department of Otolaryngology Head and Neck Surgery, Chinese PLA Institute of Otolaryngology, The Sixth Medical Center of Chinese PLA General Hospital, Medical School of Chinese PLA, 28 Fuxing Road, Beijing, 100853 People’s Republic of China; 2 National Clinical Research Center for Otolaryngologic Diseases, Beijing, 100853 People’s Republic of China; 3 Hebei Industrial Technology Research Institute of Genomics in Maternal and Child Health, Shijiazhuang, 050000 People’s Republic of China; 4 BGI Genomics, BGI-Shenzhen, Shenzhen, 518083 People’s Republic of China; 5 Department of Otolaryngology-Head and Neck Surgery, Tongji Hospital, Tongji Medical College, Huazhong University of Science and Technology, Wuhan, 430030 People’s Republic of China; 6 Medical Technology College, Hebei Medical University, Shijiazhuang, 050000 People’s Republic of China

## Abstract

Auditory neuropathy (AN) is a unique type of language developmental disorder, with no precise rate of genetic contribution that has been deciphered in a large cohort. In a retrospective cohort of 311 patients with AN, pathogenic and likely pathogenic variants of 23 genes were identified in 98 patients (31.5% in 311 patients), and 14 genes were mutated in two or more patients. Among subgroups of patients with AN, the prevalence of pathogenic and likely pathogenic variants was 54.4% and 56.2% in trios and families, while 22.9% in the cases with proband-only; 45.7% and 25.6% in the infant and non-infant group; and 33.7% and 0% in the bilateral and unilateral AN cases. Most of the *OTOF* gene (96.6%, 28/29) could only be identified in the infant group, while the *AIFM1* gene could only be identified in the non-infant group; other genes such as *ATP1A3* and *OPA1* were identified in both infant and non-infant groups. In conclusion, genes distribution of AN, with the most common genes being *OTOF* and *AIFM1*, is totally different from other sensorineural hearing loss. The subgroups with different onset ages showed different genetic spectrums, so did bilateral and unilateral groups and sporadic and familial or trio groups.

**Supplementary Information:**

The online version contains supplementary material available at 10.1007/s00439-024-02652-7.

## Introduction

Auditory neuropathy (AN), a unique form of hearing impairment, is characterized by evidence of intact outer hair cell function, accompanied by poor eighth nerve-brainstem responses (Starr A 1996), affecting 1.2–8.4% of cases with hearing loss from different populations (Foerst et al. 2006; Penido and Isaac 2013; Vignesh et al. 2016) and 0.006–0.039% in low-risk newborn population (Korver et al. 2012). The term “auditory neuropathy” was first proposed in 1996, for the patients with normal hair cell activity but abnormal auditory nerve functions (Kaga et al. 1996; Starr A 1996). As a heterogeneous disorder, AN varies in several measures including age of onset (either congenital or acquired), hereditary (either sporadic or familial), affected sites (bilateral or unilateral), presence of peripheral/optic neuropathy (either non-syndromic or syndromic), and clinical manifestation. The site of lesion causing AN may involve the presynaptic site, the synapses, and the postsynaptic site.

The diagnosis of AN mainly relies on audiological and electrophysiological testing. Previous studies of pathophysiological mechanisms of AN have shown the diagnostic criteria for presynaptic disorders (including inner hair cells and synapse disorders) and postsynaptic disorders (Rance and Starr 2015). Confusingly, patients with AN, who have similar clinical manifestations, may have different damaged anatomical site. It is difficult to distinguish dysfunction of the inner hair cells and synapses related AN from auditory nerve disorders. The precision lesion site of AN is difficult to be identified by a single pathophysiological test. It can be caused by damage of inner hair cell, synapse, auditory nerve, or the combination of these sites. Genetic testing may provide a new perspective for the diagnosis and management of AN.

Both genetic factors and environmental risk factors are attributed to AN, such as hyperbilirubinemia, anoxia, and immunological and infectious factors (Starr A 1996; Starr et al. 2000), with about 50% having no defined etiology. Thirteen genes have been reported to be related to AN, showing implications for expected cochlear implantation outcomes (Shearer and Hansen 2019), including genes that affected presynaptic site (*OTOF*), synaptic site (*CACNA1D*, *CABP2*, *SLC17A8*), postsynaptic site (spiral ganglion, *DIAPH3*, *OPA1*, *ROR1*, *ATP1A3*), and auditory nerve (spiral ganglion cell bodies and proximal axons, *TIMM8A*, *AIFM1*, *NARS2*, *MPZ*, *PMP22*). Genetic diagnosis for patients suffering from AN has critical implications for treatment, prognosis, and development of precision medicine strategies. However, to date, no large AN cohort has been built to decipher the diagnostic yield of high-throughput sequencing.

**Fig. 1:**
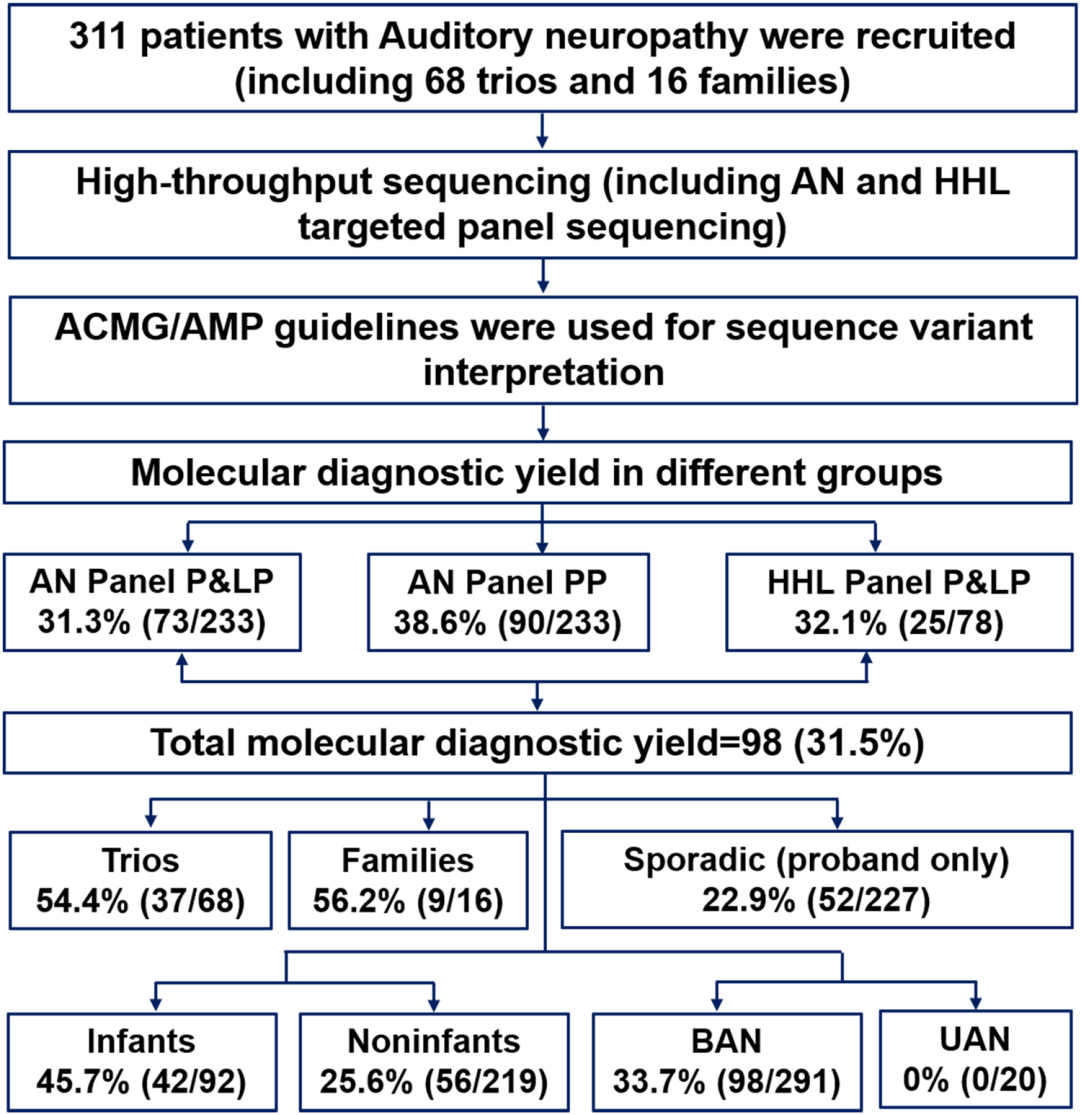
Study design and flowchart. Trios, patient with auditory neuropathy plus both parents; AN, auditory neuropathy; P, pathogenic; LP, likely pathogenic; PP, possible pathogenic; HHL, hereditary hearing loss; BAN, bilateral auditory neuropathy; and UAN, unilateral auditory neuropathy

The molecular diagnostic rate of AN varies greatly, which is supposed to be different from other genetic hearing loss, the most common attributing genes of which are *GJB2* and *SLC26A4*. Recently, genetic studies are playing an increasingly crucial role in disease diagnosis and management. Next-generation sequencing has revolutionized the discovery of rare Mendelian disease-related gene variants (Yang et al. 2014). The yield of genetic testing of AN varies based on the accompanied phenotype (syndromic and non-syndromic AN), onset age (infant and non-infant AN), affected sides, etc. In this study, we recruited 311 cases who were clinically diagnosed as AN and performed genome sequencing to detect and quantify genetic variants and assess existing therapeutic effects (Fig. 1). Our findings have implications for the future development of precision medicine strategies.

## Materials and methods

### Study participants (cases recruitment and clinical evaluations)

A total of 311 cases clinically diagnosed with AN were identified by the Institute of Otolaryngology, Chinese PLA General Hospital, between January 2003 and December 31, 2020 (Fig. 1). Personal or family medical reports were identified by a team of experienced physicians and audiologists. Audiometric evaluations included audiogram, auditory brainstem responses (ABR), distortion product otoacoustic emission (DPOAE), electrocochleography (EcochG), and speech discrimination score (SDS). Pure-tone averages (PTA) were defined as average threshold of 500, 1000, 2000, and 4000 Hz frequencies (Thorpe et al. 2021), as well as in all frequencies. Children under six years old were evaluated by auditory steady-state response (ASSR) and behavioral audiometry. The severity of hearing impairment was defined as mild (26–40 dB HL), moderate (41–55 dB HL), moderately severe (56–70 dB HL), severe (71–90 dB HL), or profound (> 90 dB HL) according to middle frequencies PTA. Cochlear microphonic (CM) was conducted including the use of inserting earphones, reverse polarity, and blocking the stimulus tube to eliminate electrical artifact interference. High-resolution computed tomography (HRCT) and/or MRI was also performed on some of the cases to verify whether the family members had complications other than hearing disorders.

Inclusion criteria were cases who were diagnosed as AN by 1) the presence of OAE and/or CM and 2) the abnormal or absence of ABR (Chinese Multi-center Research Collaborative Group on Clinical et al. 2022; Constantina Georga et al. 2019; Deborah Hayes et al. 2008; Judy Gravel et al. 2004; Mona M. Dworsack-Dodge et al. 2012; Rachel Feirn et al. 2013). Exclusion criteria were 1) acoustic neuroma and 2) cases or the guardians who refused genetic testing. For the cases with family history, only the proband was recruited in this study, and the family members were only recruited for segregation analysis, instead of phenotype analysis.

### Searching for AN-associated genes in OMIM

We used the clinical synopsis advanced search to find 494 entries for “auditory neuropathy” from Online Mendelian Inheritance in Man (OMIM), which is a database that contains all known Mendelian inheritance diseases, as well as clinical synopses that can be used to find a target gene. The following were the inclusion criteria: 1) phenotypic title includes “auditory neuropathy”, 2) clinical synopsis description includes “auditory neuropathy”, or 3) clinical aspects of reported patients include “hearing loss” or “deafness”. After that, we assembled 138 items including phenotype, gene, and inheritance information. When we combined genes from PubMed, the MGI database and common genes for genetic hearing loss, we got a total of 178 genes (Supplementary Data 1) that could be associated to AN. The genetic pattern of these 178 genes varies: 44 genes are autosomal dominant, 94 genes are autosomal recessive, 23 genes have a complicated genetic pattern that includes autosomal dominant (AD), autosomal recessive (AR), and multifactorial, and 11 genes are linked to the X chromosome.

We examined the interaction among these genes using STRING (https://www.string-db.org/), and the PPI enrichment *p*-value was less than 1.0e−16, indicating that the majority of these genes were related to one another and could form a network with substantial interactions. Outside of the network were 19 genes (*PTRH2, PORCN, RAI1, MCM3AP, TMEM43, FITM2, ADPRHL2, ORC1, ROR1, MORC2, PIGB, KIAA0586, TMEM30B, GPRASP2, SLC52A2, SNX10, TSHZ1, *and* GGT1*). To rule out the possibility of this link being random, we used random sampling to obtain three groups of genes with the same number of genes associated with AN (178). There seemed to be no substantial interaction among any of the three gene sets.

### Sequencing and bioinformatics analysis

Whole-genome sequencing (WGS, 233 cases) and clinical targeted panel sequencing (hereditary hearing loss (HHL) Panel, 78 cases) were all options for our study. The HHL Panel approach had previously been described, which focused on genes related to monogenic hearing loss (Wang et al. 2014, 2018) (Supplementary Data 2). The WGS pipeline consists of eliminating low-quality reads and adaptor reads, mapping reads to the reference genome (GRCh37) by BWA and SOAPnuke, detecting single-nucleotide polymorphisms (SNPs) and small insertion or deletion (InDel) via GATK, and finally generating a VCF file for each sample. We used VEP to annotate the detail information for SNP and small InDel containing the gene name, transcript, mutation consequence, and allele frequency in public databases (gnomAD, 1000 Genome Project, ExAC) based on the VCF file. We matched variants to all versions of a gene’s transcript depending on physical location throughout the annotation process and then output two sets of annotation results: the most severe transcript result and the authoritative transcript result. This set was to avoid missing disease-causing variants when just utilizing an authenticated transcript.

We used the following criteria to filter SNPs and small InDels from WGS data: 1) removing variants with population frequencies greater than 0.005 in the public database (gnomAD v2.1.1); 2) reserving variants located on the coding region of 178 AN-associated genes; and 3) reserving variants that may affect the structure and function of protein through consequence type (splice_acceptor_variant, splice_donor_variant, stop_gained, frameshift_variant, stop_lost, start_lost, inframe_insertion, inframe_deletion, missesnse_variant, TFBS_amplification). Following the filtering process, each sample yields fewer variants.

Then, using the standards and guidelines of the American College of Medical Genetics and Genomics (ACMG) (Oza et al. 2018; Patel et al. 2021; Richards et al. 2015), we classified the variants identified in WGS and panel sequencing as pathogenic (P), likely pathogenic (LP), variants with uncertain significance (VUS), likely benign (LB), and benign (B). Then, in two steps—true variant verification and co-segregation analysis within pedigrees—we employed Sanger sequencing to verify the variation classified as P, LP, or VUS and further classifed VUS to VUS, VUS|LP, and VUS|P. For patients with these variants, we classified the genetic testing result according to the inheritance pattern, ACMG variant classification, heterozygous or homozygous into pathogenic, likely pathogenic, possible pathogenic, carrier, and unknown (Supplementary Table 1).

### Drug gene interaction analysis

Drug-Gene Interaction database (http://dgidb.org/) was used for drug–gene interaction analysis; “search Drug-Gene Interactions” and “search Potential Druggability” were selected, respectively, to search for the known pathogenic genes of AN. All the analyses and visualizations were conducted in R 4.2.1. The following R package was used: ggplot2 package.

### Gene function analysis

Enrichment analysis was performed on the candidate genes using the database established by the Gene Ontology Consortium, focusing on biological process (BP), cellular component (CC), and molecular function (MF). All the analyses and visualizations were performed in R 4.2.1. The R package involved was clusterProfiler package (used for enrichment analysis). Sankey + dot plot was plotted by https://www.bioinformatics.com.cn (last accessed on July 10, 2023), an online platform for data analysis and visualization.

### Gene expression analysis

The expression sites of the AN new genes were analyzed using single-cell sequencing data published by van der (van der Valk et al. 2023) and Philippe Jean (Jean et al. 2023). All the data mentioned were publicly available and were analyzed online using gGAR: Gene Expression Analysis Resource portal for community-driven, multi-omic data exploration. The gene expression pattern map was created with BioRender.com.

### Cochlear implantation/hearing aid management of AN in clinic

Bilateral AN patients in this study were followed up via phone interviews, during which management strategies, aided hearing sensitivity, and speech recognition after cochlear implantation or hearing aids were collected through a unified common questionnaire. Auditory and speech abilities were evaluated by the categories of CAP and SIR (Archbold et al. 1995; Miyamoto et al. 1999; Song et al. 2021).

### Statistical analysis

Data were analyzed using the Prism 6.0 statistical analysis program (GraphPad). A two-tailed value for *p* < 0.05 was considered statistically significant. Continuous data were presented as the mean and standard deviation (SD), and categorical variables were reported as the frequency and percentage. Fisher’s exact test or chi-square test was used to assess the difference in the mutation frequencies between the persons from different subgroups of auditory neuropathy.

## Results

### Demographic features of patients

A total of 311 AN cases received high-throughput sequencing were ascertained in this study (Table 1), showing no gender preference: 47.9% (149/311) of our samples were female, and 52.1% (162/311) were male. The ethnic background of the probands was primarily Han (*n* = 285, 91.6%). The cases with newborn risk factors of jaundice or hyperbilirubinemia, infection, ototoxic drugs, and hypoxia or asphyxia were 19, 12, 12, and 9, respectively.

**Table 1:** Clinical characteristics of auditory neuropathy patients

	Total	BAN	UAN
Case numbers	311	291	20
The test age (Yrs)*		(291) 15.9 (11.0) 17.0 (3.8–23.5)	(20) 5.6 (5.2) 4.7 (1.9–6.9)
Age of onset (Yrs)*		(290) 10.9 (7.7) 12.5 (2.1–16.0)	(20) 3.7 (3.3) 4.0 (0.8–6.0)
Onset age			
≤ 3y	92	82 (28.2%)	10 (50.0%)
> 3y	219	209 (71.8%)	10 (50.0%)
Time elapse between onset and first visit (Yrs)*		(286) 5.1 (6.1) 2.4 (0.7–7.9)	(19) 2.0 (3.5) 1.0 (0.5–1.5)
Gender			
Male	162 (52.1%)	150 (51.5%)	12 (60.0%)
Female	149 (47.9%)	141 (48.5%)	8 (40.0%)
Progress (affected ears)			
Deterioration	57 (33.7%)	55 (33.9%)	2 (28.6%)
No change (or fluctuation)	80 (47.3%)	75 (46.3%)	5 (71.4%)
Improvement	32 (19.0%)	32 (19.8%)	0
Folic acid*	(90) 6.7 (4.4) 5.5 (4.1–7.9)	(90) 6.7 (4.4) 5.5 (4.1–7.9)	NA
Vitamin B12*	(91) 442.6 (409.8) 291.1 (208.2–476.6)	(91) 442.6 (409.8) 291.1 (208.2–476.6)	NA
Homocysteine acid*	(79) 23.1 (19.4) 14.9 (8.8–31.7)	(79) 23.1 (19.4) 14.9 (8.8–31.7)	NA

*BAN,* bilateral auditory neuropathy; *UAN,* unilateral auditory neuropathy; and *Yrs,* years

The onset age of AN occurred at all ages, but the typical onset age of AN was in the early childhood and during adolescence. The medium age of disease onset in this cohort was 10.9 ± 7.7 years old, ranging from 0 to 40 years old for bilateral AN, 3.7 ± 3.3 for unilateral AN. Patients were classified based on onset age: infant (0–3 years old, 92 patients, 29.6%) and non-infant (> 3 years old, 219 patients, 70.4%, including 1 case with unclear onset age, and 49 cases ≥ 18 years old); affected sides: bilateral AN (291 patients, 93.6%) and unilateral AN (20 patients, 6.4%); and family history: sporadic AN (295 patients, 94.9%) and familial AN with more than one member were diagnosed as AN in a family (16 patients, 5.1%, Supplementary Fig. 1).

Among the 291 bilateral AN cases, 4.9% (4/82) of infant AN and 65.1% (136/209) of non-infant AN complained tinnitus; 1.2% (1/82) of infant AN and 5.7% (12/209) of non-infant AN with vertigo or dizziness; 4.9% (4/82) of infant AN and 8.6% (18/209) of non-infant AN with movement disorder and numbness of limbs; 26.8% (22/82) of infant AN and 4.3% (9/209) of non-infant AN showed severe communication disorders; and 4.9% (4/82) of infant AN and 18.2% (17/209) of non-infant AN complained vision loss, among whom five cases were diagnosed of optic nerve atrophy.

### Audiological, electrophysiological, and imaging (Table 2 and Supplementary Table 2)

**Table 2:** Audiological characteristics of bilateral auditory neuropathy cases according to age

	Total	≤ 3y	> 3y	*p*-value
Progress (left ear)				0.856
Deterioration	28 (34.6%)	3 (27.3%)	25 (35.7%)	
No change (or fluctuation)	34 (42.0%)	5 (45.5%)	29 (41.4%)	
Improvement	19 (23.5%)	3 (27.3%)	16 (22.9%)	
Progress (right ear)				0.384
Deterioration	27 (33.3%)	2 (18.2%)	25 (35.7%)	
No change (or fluctuation)	41 (50.6%)	6 (54.5%)	35 (50.0%)	
Improvement	13 (16.0%)	3 (27.3%)	10 (14.3%)	
Threshold assessed by pure-tone or visual reinforcement audiometry				
Left ear				
250 Hz	(225) 59.3 (19.3) 60.0 (45.0–70.0)	(22) 87.0 (19.3) 92.5 (81.2–95.0)	(203) 56.3 (16.8) 55.0 (45.0–70.0)	< 0.001
500 Hz	(231) 60.0 (22.8) 60.0 (45.0–70.0)	(28) 95.4 (19.4) 100.0 (90.0–106.2)	(203) 55.2 (18.5) 55.0 (45.0–65.0)	< 0.001
1 kHz*	(232) 52.3 (26.4) 50.0 (35.0–65.0)	(29) 92.4 (21.1) 95.0 (90.0–105.0)	(203) 46.6 (21.7) 45.0 (30.0–60.0)	< 0.001
2 kHz*	(231) 40.8 (28.4) 30.0 (20.0–55.0)	(28) 87.1 (23.8) 87.5 (80.0–105.0)	(203) 34.5 (22.4) 30.0 (20.0–40.0)	< 0.001
4 kHz*	(230) 39.6 (29.2) 30.0 (20.0–55.0)	(28) 88.2 (26.1) 82.5 (70.0–106.2)	(202) 32.8 (22.4) 25.0 (20.0–45.0)	< 0.001
8 kHz*	(222) 39.7 (28.4) 30.0 (20.0–55.0)	(20) 84.2 (21.8) 85.0 (70.0–101.2)	(202) 35.3 (25.0) 27.5 (15.0–50.0)	< 0.001
PTA in 0.5,1,2,4 kHz	(232) 48.5 (24.9) 41.9 (32.5–57.8)	(29) 91.2 (20.5) 91.2 (82.5–105.0)	(203) 42.4 (18.7) 40.0 (30.0–51.2)	< 0.001
PTA in all frequency	(232) 49.4 (23.5) 41.7 (33.3–59.0)	(29) 90.3 (18.6) 91.7 (82.5–104.2)	(203) 43.5 (17.7) 40.0 (32.5–52.9)	< 0.001
Right ear				
250 Hz	(225) 60.0 (20.1) 60.0 (45.0–70.0)	(22) 88.0 (26.7) 95.0 (80.0–110.0)	(203) 56.9 (16.8) 60.0 (45.0–70.0)	< 0.001
500 Hz	(231) 60.3 (22.2) 60.0 (45.0–70.0)	(28) 90.4 (24.8) 97.5 (83.8–105.0)	(203) 56.2 (18.3) 60.0 (45.0–70.0)	< 0.001
1 kHz*	(233) 53.6 (27.2) 50.0 (35.0–70.0)	(30) 93.2 (26.7) 100.0 (90.0–110.0)	(203) 47.8 (21.9) 50.0 (32.5–60.0)	< 0.001
2 kHz*	(232) 42.6 (29.2) 35.0 (20.0–60.0)	(29) 90.0 (28.1) 90.0 (80.0–110.0)	(203) 35.8 (22.2) 30.0 (20.0–45.0)	< 0.001
4 kHz*	(231) 42.3 (29.3) 30.0 (20.0–60.0)	(28) 88.0 (29.7) 90.0 (72.5–110.0)	(203) 35.9 (23.1) 30.0 (20.0–45.0)	< 0.001
8 kHz*	(223) 38.5 (27.2) 30.0 (20.0–57.5)	(20) 78.0 (27.0) 77.5 (65.0–100.0)	(203) 34.6 (24.0) 30.0 (15.0–50.0)	< 0.001
PTA in 0.5,1,2,4 kHz	(233) 50.0 (25.2) 45.0 (32.5–58.8)	(30) 91.4 (25.2) 95.0 (85.0–107.2)	(203) 43.9 (18.7) 42.5 (31.2–53.8)	< 0.001
PTA in all frequency	(233) 50.4 (24.0) 45.8 (35.0–60.0)	(30) 90.4 (24.4) 95.0 (84.4–105.0)	(203) 44.5 (17.4) 42.5 (33.7–52.9)	< 0.001
Elicited ABR				
Left ear	21 (7.2%)	3 (3.7%)	18 (8.6%)	0.142
Right ear	27 (9.3%)	3 (3.7%)	24 (11.5%)	0.038
Presence of CM				< 0.001
Left ear	118 (40.5%)	65 (79.3%)	53 (25.4%)	
Right ear	123 (42.3%)	66 (80.5%)	57 (27.3%)	
Presence of DPOAE				
Left ear				
0.5 kHz	102 (57.6%)	18 (32.1%)	84 (69.4%)	< 0.001
0.75 kHz	193 (68.7%)	25 (31.2%)	168 (83.6%)	< 0.001
1 kHz	209 (73.1%)	27 (32.9%)	182 (89.2%)	< 0.001
1.5 kHz	213 (75.8%)	35 (43.8%)	178 (88.6%)	< 0.001
2 kHz	224 (78.3%)	43 (52.4%)	181 (88.7%)	< 0.001
3 kHz	223 (79.1%)	57 (70.4%)	166 (82.6%)	0.022
4 kHz	223 (78.0%)	54 (65.9%)	169 (82.8%)	0.002
6 kHz	216 (76.1%)	53 (65.4%)	163 (80.3%)	0.008
8 kHz	196 (72.3%)	49 (59.8%)	147 (77.8%)	0.002
Right ear				
0.5 kHz	107 (59.1%)	20 (34.5%)	87 (70.7%)	< 0.001
0.75 kHz	187 (66.5%)	23 (28.7%)	164 (81.6%)	< 0.001
1 kHz	219 (76.8%)	34 (41.5%)	185 (91.1%)	< 0.001
1.5 kHz	227 (80.8%)	43 (53.8%)	184 (91.5%)	< 0.001
2 kHz	232 (81.4%)	51 (62.2%)	181 (89.2%)	< 0.001
3 kHz	231 (81.9%)	58 (71.6%)	173 (86.1%)	0.004
4 kHz	228 (80.3%)	59 (72.0%)	169 (83.7%)	0.025
6 kHz	219 (77.4%)	57 (70.4%)	162 (80.2%)	0.074
8 kHz	205 (75.6%)	50 (61.0%)	155 (82.0%)	< 0.001

Variable representation method: (N), mean; (SD), median (Q1–Q3)*; PTA,* pure-tone average; *ABR,* auditory brainstem responses; *CM,* cochlear microphonic; *DPOAE,* distortion product otoacoustic emission; and *ASSR,* auditory steady-state response

Pure-tone thresholds ranged from normal to total deafness, with the PTA in all frequencies 49.9 dB HL. The PTA in all frequencies of the patients in infant group and non-infant group were 90.4 and 44.0 dB HL, respectively. The audiograms were various, and the most common type was ascending in non-infant group and total deaf in infant group. Most of the bilateral AN cases (81%) had symmetrical audiograms (Supplementary Fig. 2). A total of 298 ears had speech audiometry data in the first consultation, the PTA was 42.13 ± 18.65 dB HL, and the SDS was 40.27 ± 30.07%. There was a significant correlation between speech recognition rate and average hearing threshold (*p* = 0.001, Supplementary Fig. 3). To compare the correlation between the pure-tone hearing threshold of each frequency or the ASSR threshold of each frequency and the speech recognition rate, the ASSR (especially 1 kHz on the left) may have a better correlation with speech recognition rate, from the perspective of the correlation coefficient for bilateral AN (Supplementary Fig. 3).

The elicitation rate of DPOAE in different frequencies ranged from 57.6 to 81.95%, and the 500 Hz had the lowest elicitation rate. The overall elicitation rate in infant group was lower than non-infant group, and the low frequency (0.5–1.5 kHz) extraction rate was less than half; while in the non-infant group, the extraction rate was relatively low in 500 Hz (Supplementary Fig. 4). A total of 241 ears could record CM waves, and the presence rate in infant and non-infant group was 79.9% and 26.4%, respectively. For ABR testing, most ears (92.4%, 556/602) did not elicit a response at the maximum sound intensity (> 100 dBnHL), and 46 ears (7.6%) could elicit ABR waveforms, but the waveform differentiation was severely abnormal, with three ears (6.5%) only showing I wave, four ears (8.7%) eliciting I.III.V wave, but the amplitudes were abnormal, and the remaining 39 ears (84.8%) only had V wave. A total of 244 ears were conducted electrocochleogram, 17 ears showed no response, and 1 ear only recorded action potential (AP), 28 ears only recorded summing potential (SP), the SP/AP ratios were available in 198 ears, with 20 ears < 0.6, 67 ears ranged from 0.6 to 1, and 111 ears > 1, respectively. Audiological and electrophysiological features of the affected ears of unilateral AN were shown in Supplementary Data 3.

Fifty-five probands received temporal CT, and most of them showed no obvious abnormalities. Ninety-five cases, including 12 unilateral auditory neuropathy (UAN) cases and 83 bilateral auditory neuropathy (BAN) cases, were performed MRI scanning, among which 25 cases (26.3%, 25/95) showed unilateral or bilateral auditory nerve abnormalities, including slender, unclear, or missing auditory nerves. Among the 25 cases with abnormal MRI, six cases were unilateral AN, and 19 cases were BAN.

### Following-up

For patients who have visited our hospital more than two times (139 cases in total), the audiological testing of the latest and the first time was compared. The average follow-up period was 42.84 ± 4.54 months, ranging from 1 to 301 months. The middle-frequency PTA was used to evaluate the progression of the affected ears, and PTA increase more than 10 dB, change ≤ 10 dB, and decrease more than 10 dB were determined as deterioration, no change (or fluctuation), and improvement. The deterioration, no change, and improvement ears were 57 (33.7%), 80 (47.3%), and 32 (19.0%), respectively. The progression showed no difference between infant and non-infant groups. Thirty-two cases (63 ears) with the following SDS were analyzed, with a follow-up period of 47.03 ± 12.87 months, ranging from 1 to 301 months. There was no significant difference between the first and last SDS, the average scores of which were 28.16 ± 2.953%, and 32.86 ± 3.237%, respectively.

### Genetic spectrum in this AN cohort

For the WGS data from 233 cases, we had a total of 541 candidate disease-related SNPs and InDel located on 138 genes after the selection process, including variants classified as VUS, LP, and P. A total of 69 variants were shared by two or more samples, and 472 variants appeared only once. A total of 200 AN patients had these variants.

If more than two patients have the same genotype, the evidence for a link between genotype and phenotype is higher. Further, according to ACMG variant classification, inheritance pattern, and heterozygous or homozygous type, we classified the variants as carrier, unknown, possible pathogenic, likely pathogenic, and pathogenic variants from the perspective of subjects instead of variants only (Supplementary Table 1). A total of 90 cases (38.6%, 90/233) were identified with possible pathogenic variants, and 73 cases (31.3%, 73/233) were identified with pathogenic or likely pathogenic results. For the cases with pathogenic or likely pathogenic variants, the top gene was *AIFM1*, which had likely pathogenic or pathogenic variations in 23 samples, followed by *OTOF*, which had compound heterozygous variations in 17 of the samples. *MYO7A* (nine samples), *TWNK* (seven samples), *WFS1* (six samples), and *ATP1A3* (five samples) were the next four genes. More than five samples shared each of these six genes. Totally 142 cases were carriers, with 233 variants of 68 recessive genes, the top three genes were *SLC52A2*, *SLC52A3*, and *SLC26A4*, with 18, 15, and 14 carriers, respectively.

Eleven genes were identified by both of the methods, including *AIFM1*, *ATP1A3*, *FDXR*, *GJB2*, *MYO7A*, *NARS2*, *OPA1*, *OTOF*, *SLC17A8*, *SLC26A4*, *TIMM8A*, *TSPEAR,* and *WFS1*. Among these genes, likely pathogenic and pathogenic variants were identified in *AIFM1* (5 samples), *ATP1A3* (2 samples), *OPA1* (4 samples), *OTOF* (12 samples), *TIMM8A* (1 sample) from the panel sequencing.

Cross WGS and panel sequencing methods, 73 cases (31.3%, 73/233) by WGS methods and 25 cases (32.0%, 25/78) from panel sequencing with likely pathogenic or pathogenic variants were identified. In summary, the eight most common genes in our cohort were *OTOF* (29 samples), *AIFM1* (28 samples), *MYO7A* (9 samples), *ATP1A3* (7 samples), *WFS1* (6 samples), *OPA1* (5 samples), *NOTCH3* (4 samples), and *TWNK* (4 samples) (Table 3). For the most common gene in hearing loss population, one case had *GJB2* compound heterozygous mutation (1,007,133), with infant onset bilateral profound hearing loss and presence of otoacoustic emission (OAE), and two cases found *GJB2* heterozygous mutation (1,507,400 and 1,006,855).

**Table 3:** Genes with pathogenic and likely pathogenic variants identified in AN cohort

Gene	RefSeq accession No	No. of cases		Total	Inheritance
		Infant	Non-infant		
*AIFM1*	NM_004208.4	0	28	28	XLR
*OTOF*	NM_001287489.2	28	1	29	AR
*MYO7A*	NM_000260.4	4	5	9	AD, AR
*ATP1A3*	NM_152296.5	2	5	7	AD
*WFS1*	NM_006005.3	3	3	6	AD, AR
*OPA1*	NM_130837.3	3	2	5	AD, AR
*NOTCH3*	NM_000435.3	2	2	4	AD
*TWNK*	NM_021830.5	1	3	4	AD, AR
*GJB2*	NM_004004.6	1	0	1	AD, AR
*KIF5A*	NM_004984.4	1	1	2	AD
*NEFL*	NM_006158.5	1	1	2	AD, AR
*TIMM8A*	NM_004085.3	0	2	2	XLR
*CDH2*	NM_001792.5	0	2	2	AD
*SLC52A3*	NM_001370086.1	0	2	2	AR
*CACNA1A*	NM_023035.3	1	0	1	AD
*FDXR*	NM_001258012.4	0	1	1	AD, AR
*GREB1L*	NM_001142966.3	1	0	1	AD
*PRX*	NM_181882.3	1	0	1	AD, AR
*MFN2*	NM_014874.4	0	1	1	AD, AR
*NF2*	NM_022489.4	0	1	1	AD
*SMAD4*	NM_005359.6	1	0	1	AD
*TP63*	NM_003722.5	0	1	1	AD
*TRPV4*	NM_021625.4	1	0	1	AD

*AD,* autosomal dominant; *AR,* autosomal recessive; and *XLR,* X-linked recessive

**Fig. 2:**
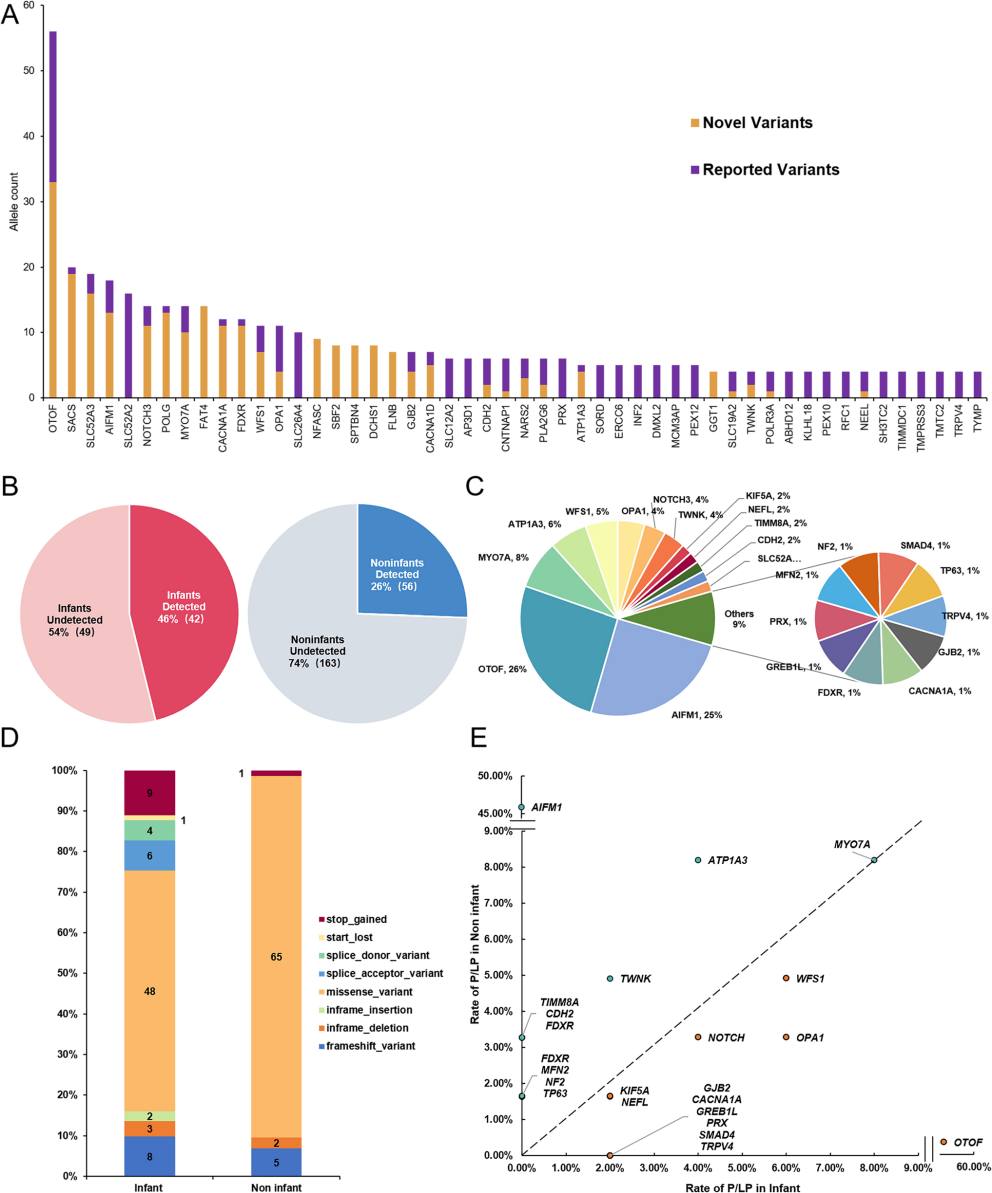
Overview of the genetic spectrum in the AN cohort. **A** Distribution of the top 50 genes of the number of sites, including both novel and reported variants. **B** The diagnostic rates for infant and non-infant groups. **C** Genes contribution of the 98 patients with P/LP variants. **D** The mutation type of P/LP variants in cases of infant and non-infant groups. **E** The prevalence of P/LP variants in cases of infant and non-infant groups. P/LP, pathogenic or likely pathogenic

Collectively, 584 candidate variants in 139 genes of 243 cases were identified to be P, LP, or VUS, accounting for 11.6% (68/584), 15.9% (93/584), and 72.4% (423/584), respectively. The distribution of the top 50 genes can be seen in Fig. 2A, based on the number of variants and whether they were novel or reported. Previously reported variants were those identified to be damaging according to ClinVar or published studies.

A total of 154 variants spanning 23 genes were identified and classified as pathogenic or likely pathogenic in 98 patients (31.5%) (Fig. 2C and Supplementary Data 4). Among them, 73/154 variants (47.4%) were previously reported as pathogenic, and 81/154 variants (52.6%) were novel. Among the 154 variants, 113 were missense variants, 13 were frameshift variants, 10 were stop_gained variants, 9 were splicing variants, 7 were inframe variants, and 1 was start_lost variant (Fig. 2D). Further categorization of the genotypic and phenotypic data of 98 patients with confirmed pathogenic genes was conducted (Supplementary Data 5).

**Fig. 3:**
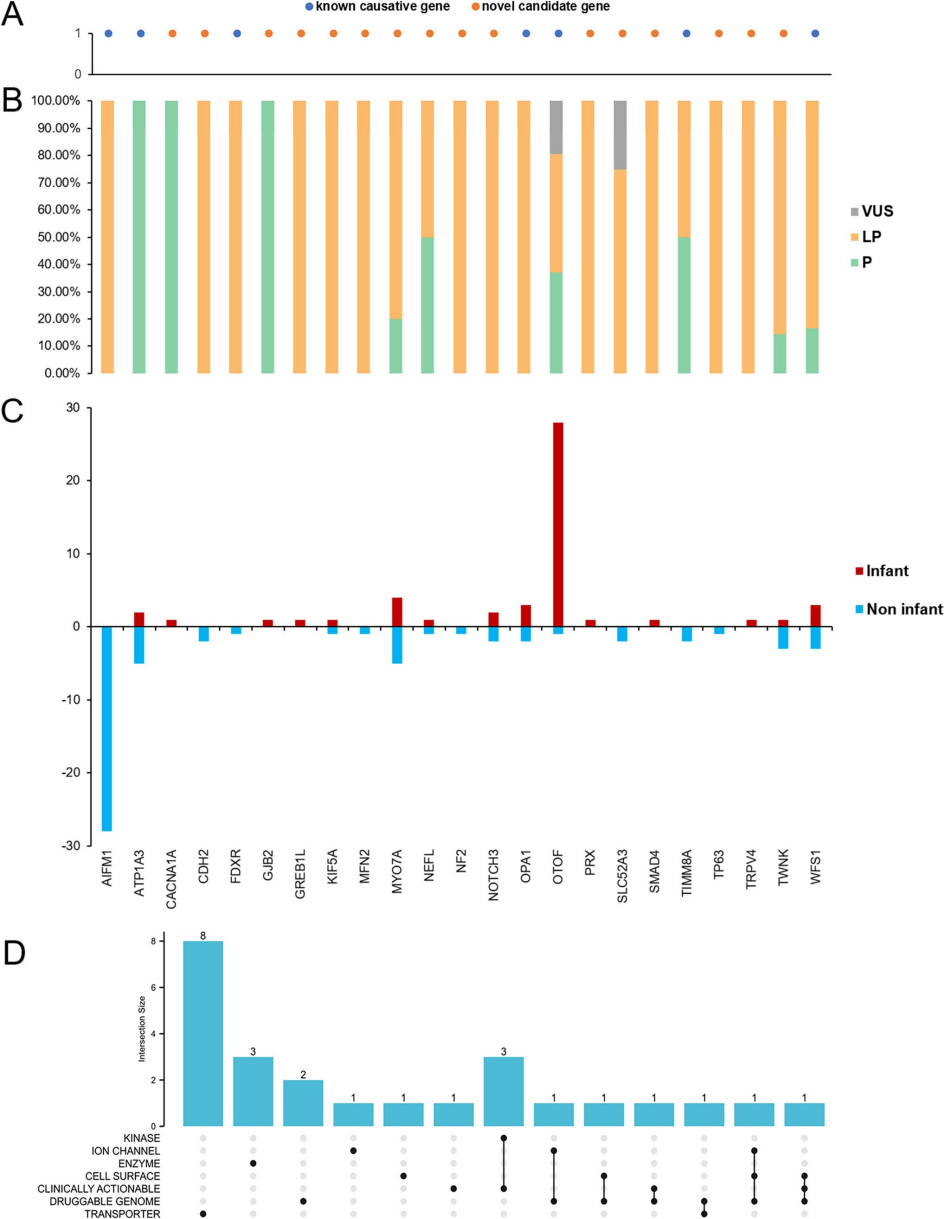
Overview of genes with P/LP variants. **A** The genes into known causative genes and novel candidate genes. **B** The P/LP/VUS ratio of each gene. **C** The number of gene sites in infant/non-infant groups. **D** The upset plot of potential druggability of each gene. P, pathogenic; LP, likely pathogenic; and VUS, variants with uncertain significance

Among the 23 genes with pathogenic or likely pathogenic variants, 7 genes were known AN causative genes, and 16 genes were novel candidate genes (Fig. 3A). The P/LP/VUS ratio of each gene and the number of gene sites in infant/non-infant groups were shown in Fig. 3B and C. Furthermore, the Drug-Gene Interaction database (DGIdb) was used to search for drug–gene interactions and potential druggability (Supplementary Data 6, Supplementary Table 3). The results of potential druggability were visualized in an upset plot (Fig. 3D).

The mode of inheritance was autosomal recessive for 30 cases, autosomal dominant for 29 cases, X-linked for 26 cases, and 13 cases with more than two diagnoses (Table 4).

**Table 4:** Inheritance pattern of 98 (31.5%) AN patients with molecular diagnosis

Inheritance	Allele transmission	Number of diagnoses	%	Total %
Autosomal dominant	De novo	7	24.14	29.59
	Inherited	11	37.93	
	Inherited unknown	11	37.93	
Autosomal recessive	Compound heterozygous	26	86.67	30.61
	Homozygous	1	3.33	
	Phase unknown	3	10.00	
X-linked hemizygous	Carrier mother/de novo	26	26.53	26.53
Two diagnoses	Autosomal dominant + autosomal dominant	5	38.46	13.27
	Autosomal dominant + autosomal recessive	3	23.08	
	Autosomal dominant + X-linked	3	23.08	
	Autosomal recessive + autosomal recessive	0	0.00	
	Autosomal recessive + X-linked	1	7.69	
	Autosomal dominant + autosomal dominant + autosomal recessive	1	7.69	

### Genetic spectrum in different subgroups

Testing of probands concurrently with parents (trio; *n* = 68) significantly improved the molecular diagnostic yield from 22.9% (52/227, proband-only) to 54.4% (37/68) (chi-square test, *p* < 0.05). Among trios with positive diagnostic results (*n* = 37), 64.9% (24/37) were inherited in an autosomal recessive manner, 25.0% (9/36) were inherited in an autosomal dominant manner, including six autosomal dominant de novo variants, and 11.1% (4/36) were X-linked. Testing of families with more than one patient also improved the molecular diagnostic yield, 56.2% (9/16) families were identified as likely pathogenic or pathogenic variants, including five X-linked (*AIFM1* gene), two autosomal dominant (*OPA1* gene), and two autosomal recessive (*OTOF* gene) inheritance.

The subgroups with different onset ages of hearing loss showed different variants spectrum. The diagnostic rate in the infant group was 45.7% (42/92), which was significantly higher than non-infant patients who had disease onset more than 3 years old (25.6%, 56/219) (Fig. 2B). The diagnostic rates of the 23 genes with P or LP variants between infant and non-infant groups were plotted (Fig. 2E). In total, we identified 50 likely pathogenic and pathogenic variants in *OTOF*, only one patient with AN onset within the non-infant age, and the distribution showed a significant difference. We identified 13 variants in *AIFM1* gene, no (0%) patients with AN in the infant period, and the distribution showed a significant difference (*p* < 0.0001).

No pathogenic or likely pathogenic variants were identified in the cases with unilateral auditory neuropathy.

### Gene function and expression analysis

**Fig. 4:**
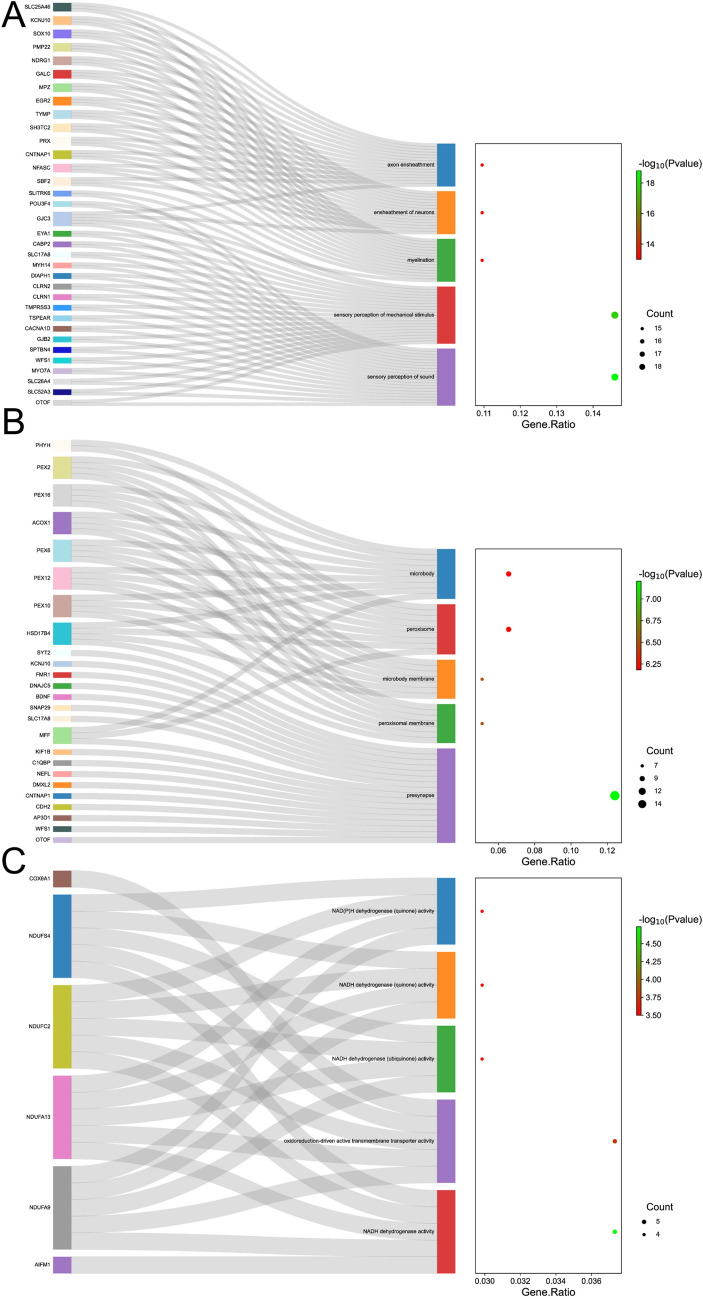
Genes function clustering analysis of the 139 genes. **A** The top 5 biological processes of the 139 genes. **B** The top 5 cell components of the 139 genes. **C** The top 5 molecular functions of the 139 genes

Gene function clustering analysis was performed for biological process, cell component, and molecular function on the 139 genes of 584 candidate variants (Fig. 4 and Supplementary Data 7). The top 5 biological processes were sensory perception of sound, sensory perception of mechanical stimulus, myelination, ensheathment of neurons, and axon ensheathment. The top 5 cell components were presynapse, peroxisomal membrane, microbody membrane, peroxisome, and microbody. The top 5 molecular functions were NADH dehydrogenase activity, oxidoreduction-driven active transmembrane transporter activity, NADH dehydrogenase (ubiquinone) activity, NADH dehydrogenase (quinone) activity, and NAD(P)H dehydrogenase (quinone) activity.

In this study, a total of 34 genes with pathogenic, likely pathogenic, or possible pathogenic variants were defined as novel AN genes (Supplementary Table 4). The expression analysis of these novel AN genes was performed using the single-cell sequencing data of the human cochlea published by van der (van der Valk et al. 2023). A comparative heatmap of the expression between 9.2-week-old infants (cochlea not fully differentiated) and adult human cochlea at the single-cell level was presented in Supplementary Fig. 5, to explore the expression patterns of the new genes during different stages of cochlear development. Further analysis was made on the expression of AN new genes in different cells of the cochlea for the data of adult human cochlea, and the results were shown in Supplementary Fig. 5B.

Since the data analyzed in van der et al.’s article were classified according to the cochlea at the developmental stage, the cells such as inner and outer hair cells of cochlea could not be distinguished. Further, using the single-cell sequencing results of mouse cochlea published by Philippe Jean (Jean et al. 2023), detailed cell type expression analysis of the expression location of the novel AN gene was performed. The expression of different cell types in P8 and P20 mice was analyzed, respectively, and the results were shown in Supplementary Fig. 6A and B and were further drawn into the gene expression pattern map, as shown in Supplementary Fig. 6C and D.

### Cochlear implantation interventions and effectiveness

A total of 136 bilateral AN cases were followed up successfully via phone review (a unified common questionnaire, Supplementary Table 5), among which 41 cases (30.15%, 41/136) in this study received cochlear implantation (CI), the surgery age of who ranged from 10 months to 342 (336) months. Of the 41 cochlear implant recipients, 4 cases (9.76%, 4/41) had bilateral implants, 19 cases (46.34%, 19/41) had monaural implants (6 left, 13 right), and 18 cases (43.90%, 18/41) had a hearing aid with a cochlear implant (bimodal hearing). The follow up of intervention was conducted in 41 patients, and the intervention duration was 5.98 ± 3.62 years. Eighty-five patients had no intervention of hearing aid or CI, and 10 patients received hearing aids only. The medium points of categories of auditory performance (CAP) scores in the CI group, CI + hearing aid (HA) group, HA group, and no intervention group were 6 (1–7), 6 (5–6), 5 (4–7), and 5 (0–7), respectively (Supplementary Fig. 7). The medium points of speech intelligibility rating (SIR) score in the CI group, CI + HA group, HA group, and no intervention group were 4 (0–5), 4 (2–5), 3 (2–5), and 5 (1–5), respectively (Supplementary Fig. 7). There was no difference in CAP and SIR scores among different intervention models. Cochlea implant outcome in cases with genetic diagnosis showed in Supplementary Table 6.

## Discussion

To the best of our knowledge, this is the largest study interrogating the genetic spectrum in cases with AN, and this cohort study first demonstrates the genetic spectrum in different subgroups of AN, especially the different onset ages. Previous genetic studies of AN focused on cases with one gene based on families, case series, or single case report. In our cohort, AN can be unilateral or bilateral, congenital infant or acquired non-infant, and producing all degrees of hearing loss. Genes distribution of AN is totally different from sensorineural hearing loss, with different molecular diagnostic yields by different targeted genes analysis and in different phenotypic subgroups.

### Phenotypic characteristics of the cohort

Most cases (93.6%) showed bilateral auditory neuropathy, and fewer cases (6.4%) showed unilateral AN, with another side presenting normal hearing or sensorineural hearing loss. Among the 20 unilateral AN cases recruited in this study, twelve cases received MRI scanning, and six cases showed cochlear nerve deficiency (CND) (50.0%, 6/12), and relatively rare genetic causes were identified.

Hearing abilities between infant and non-infant AN groups were different, infant AN showing much more severe hearing loss. Hearing threshold showed great individual differences in patients with AN, and the hearing of some cases may be improved, may remain stable for a long time, and may further deteriorate during follow-up period. Averagely, the pure-tone average threshold was stable, but some cases showed progressive worsening hearing ability involving loss of OAEs over time, as well as progressive decreased in speech recognition scores. The PTA and the SDS in quiet varied so great, and neither of them can provide significant information for the evaluation of AN. Only 7.6% of ears in our cohort could elicit at least one wave, lower than the previous study of 25% (Berlin et al. 2010).

The EcochG potential was composed of three components, including CM, SP, and AP. Most ears (81.1%) in our group showed both SP and AP, and the SP/AP ratios were > 1 for more than half ears. In our cohort, EcochG of 244 ears was recorded, which is hard to classify to the three modes being described in the previous study to locate the affected lesion side (Santarelli et al. 2008). Studies have shown that EcochG, which measures cochlear and early neural responses as acoustic signals are transmitted from cochlear to the brain, may have the role of locating the lesions of auditory neuropathy. CM waves were recorded in 241 ears (77.5%); in addition to proving the existence of outer hair cell function, CM may also be used to speculate the location of AN lesions (Shi et al. 2012).

A total of 26.3% of AN cases showed auditory nerve abnormalities in MRI, including 6 unilateral and 19 bilateral cases. In the cases with cochlear nerve deficiency, about 10% showed possible AN with the presence of DPOAE (Matsuura et al. 2021). The previous study showed that most UAN cases were the result of auditory nerve hypoplasia (Buchman et al. 2006); in our cohort, 50% (6/12) of UAN cases showed CND, while another 50% showed normal cochlear nerve, showing no difference with our previous data (38.89%, 7/18) (Song et al. 2021).

### Genotype analysis of AN

High-throughput sequencing in AN patients identified pathogenic and likely pathogenic variants with a prevalence of 31.5%. Most AN subjects were sporadic cases with no family history (94.9%, 295/311), and the inheritance pattern include de novo, autosomal dominant, AR, and X-linked. For patients with family history and trio cases concurrently with parents, the diagnostic rate of likely pathogenic and pathogenic variants were 56.2% and 54.4%, higher than the proband-only testing results. To improve the molecular diagnostic rate, we could perform follow-up of the proband-only patients. More than one hundred cases were carriers in our cohort, who can acquire information about whether they have an increased risk of conceiving a child affected with an autosomal recessive or X-linked condition (Sparks 2020; Van Steijvoort et al. 2020). In addition, the analysis of copy number variants for the cases carries one pathogenic and likely pathogenic variants in future may provide much more molecular information for AN, and the underlying mechanism of digenic inheritance of some cases in this cohort.

There were 9 genes that were mutated in single cases and 14 genes that had pathogenic and likely pathogenic variants in two or more patients. The identification of genes mutated in two or more cases provided strong evidence for their role in AN. When genetic counseling was provided, the second pathogenic variant should be taken into consideration. *OTOF* and *AIFM1* are the top two common genes in our cohort, the sites of which are presynaptic and postsynaptic lesions, affecting infant and non-infant AN cases, respectively. *OTOF* is the most common gene in our infant subgroup, previous studies showed that biallelic *OTOF* variants cause congenital or early onset (*n* = 114) hearing impairment mainly, and only few variants have been identified with progressive hearing loss (*n* = 3) (Vona et al. 2020). Gene therapy has achieved improvement in AN mouse models, and research studies have rescued partial hearing of *Otof*^−/−^ mouse by single-AAV or dual-AAV mediated system (Akil et al. 2019; Al-Moyed et al. 2019; Rankovic et al. 2020), and further leading to long-lasting hearing reversal by intein-mediated protein recombination (Tang et al. 2022). *AIFM1* is the most common gene in our non-infant subgroup, and pathogenic variants in *AIFM1* have also been identified in cases with combined oxidative phosphorylation deficiency 6, Cowchock syndrome, and spondyloepimetaphyseal dysplasia. Patients with *AIFM1* mutations are supposed to have poor effects from cochlear implantation, and the functional studies of its role in caspase-independent cell death and mitochondrial metabolism may provide potential therapy targets for *AIFM1*-related AN. *AIFM1* is the most common gene in the non-infant subgroup. As far as we are concerned, the disruption of AIFM1 may have a cumulative effect on impairing hearing function and pathogenic auditory pathways. When the auditory pathways are exposed to external sounds, noise, or other forms of negative stress, there is a gradual impairment in normal function, which is manifested as delayed progressive AN.

Exome sequencing, especially HHL-related gene panel, is currently used as a first-tier diagnostic test for individuals with hearing loss. The panels cover most of the genes of HHL, including some of the AN-related genes. The major genetic causes of integral hearing loss are *GJB2* and *SLC26A4* (Patel et al. 2021), while specific AN is totally different. *GJB2* is the most common gene in non-syndromic hearing loss population, and the c.235delC mutation in *GJB2* is the most frequently known mutation in some East Asian groups, with a carrier frequency of approximately 1% (Wang et al. 2021a). However, in our study group with AN, only one pair of compound heterozygous mutation and two heterozygous mutations were identified, with no c.235delC. Previous studies showed some patients with *GJB2* mutations were audiological confirmed AN (Cheng et al. 2005; Santarelli et al. 2008). It is still confusing and unclear how *GJB2* mutation selectively affects the afferent compartment of the cochlea but leaving unchanged out hair cell function (normal OAE and/or CM); further research is needed to clarify the *GJB2* genotype and AN phenotype correlation. For the *SLC26A4*, 5.8% (18/311) cases in our group were carriers, with no definite molecular diagnosis.

Through functional analysis, we found that the biological process of the candidate pathogenic genes in AN was mainly concentrated on sensory perception of sound and sensory perception of mechanical stimulus. Cell components were mainly concentrated in presynapse and peroxisomal membrane. Molecular function was mainly concentrated in NADH dehydrogenase activity and oxidoreduction-driven active transmembrane transporter activity. These results indicate that the pathogenic mechanism of AN is mainly concentrated in the upstream and downstream of neuronal synapses, providing evidence for the molecular biological function and selection of therapeutic targets for AN. Then, the potential drug targets and possible effective medicine were found in transporter, druggable genome, and clinically actionable categories through the analysis of the drug associations of AN pathogenic genes, providing evidence for further drug treatment of AN.

By analyzing the expression sites of AN new genes in the cochlea, it was found that the genes are expressed in different developmental stages of the cochlea, and the expression cells are more diverse in non-infants. The expression sites are mainly concentrated in type I neurons and type II neurons. Our findings provide new evidence for the classification of gene expression sites in AN.

### Management and consultation of AN

The identification of pathogenic and likely pathogenic variants in AN cases may result in genomics-informed changes in clinical care for some patients. Genetic diagnosis may provide a better understanding of affected lesions; positive results were identified in *OTOF*, *CACNA1D*, *OPA1*, *ATP1A3*, *TIMM8A*, *PMP22,* and *NARS2* in this study. Among these genes, *OTOF* and *CACNA1D* are synapse-related genes, *OPA1* and *ATP1A3* are postsynaptic spiral ganglion-related genes, and patients with pathogenic mutations of these genes are supposed to have significant effects from CI. The effectiveness of CI in patients with *OTOF* gene mutations has been reported in more than 60 cases (Zheng and Liu 2020). While *TIMM8A*, *PMP22,* and *NARS2* are postsynaptic auditory nerve-related genes, patients with these gene mutations may receive few benefits from CI. In addition, some patients had variants in genes previously associated with syndromic hearing loss, and AN may be a biomarker for neurodegenerative disease, as the first symptom of patients with neurodegenerative conditions (Rance and Starr 2015; Starr A 1996), such as Friedreich ataxia and Charcot-Marie-Tooth disease, CAPOS (Wang et al. 2021b), MTS (Wang et al. 2019), et al.

The CI results are an area of controversy for AN, which is unpredictable. Since the first report on the implantation of CI in AN patients in 1999 (Miyamoto et al. 1999), many studies have shown effective CI results in AN patients (Daneshi et al. 2018; Shearer and Hansen 2019). In general, CI improves the pure-tone threshold and the speech recognition score (Lin et al. 2022), but there is general agreement that outcomes are poor in patients with an absent cochlear nerve or severely narrowed internal auditory canal (Papsin 2005). The exact sites of the lesion of AN can determine the CI performance. Lesions located in the presynaptic and dendrite are associated with good CI performance, while the lesions in the ganglion cells and proximal axon are not. For example, patients with *OTOF* homozygous or compound heterozygous mutations are supposed to have good CI outcomes, while patients with *AIFM1* or *TIMM8A* mutations are supposed to have poor CI results. Since patients with *AIFM1* or *TIMM8A* mutation in this study showed no severe or profound hearing impairment, CIs were not chosen by the patients or the guardians.

There are some limitations in this study, indicating the future study directions of AN genotype and phenotype correlation. First, different instead of consistent sequencing strategies were performed on the patients; however, all variants were analyzed and evaluated for pathogenicity using standards guidelines. In this study, we focused on the AN-related genes for analyzing WGS data and HHL-related genes for panel data and further compared the different positive rates of these two methods. Second, some cases in this cohort have not available parental samples to assess for variant inheritance, resulting in a conservative result since some do novo variants may be missed. Third, we did not discuss the subgroups of non-syndromic or syndromic AN in this study, since the presence of peripheral/optic neuropathy and the presence of auditory neuropathy were not present at the same time, and the phenotypes of peripheral/optic neuropathy were not complete in this study. We will perform a follow-up study to decipher the constitution of non-syndromic and syndromic AN. Fourth, we conducted a retrospective analysis on the effect of CI in different genetic AN patients, and some of the cases who have received cochlear implantation could not be followed. We will perform a forward-looking study based on the genetic results in this study, to find out the CI effect of patients with different genes, which are located in different sites, providing a precise treatment strategy to the patients.

In summary, we present the first large study of comprehensive high-throughput sequencing and genotype–phenotype correlation analysis in a Chinese AN cohort, identifying the genetic spectrum of AN cases and deciphering that the subgroups with different onset ages showed a diverse genetic spectrum of AN cases. Further, the molecular diagnostic rate of targeted AN genes and targeted HHL genes was 69.9% and 32.1%, respectively. Molecular advances in understanding the various forms of AN will guide us toward more precise management strategies and abilities to predict outcomes. Further research is needed to understand the clinical implications of these findings.

### Supplementary Information

Below is the link to the electronic supplementary material.

Supplementary file1 (XLSX 113 KB)

Supplementary file2 (DOCX 3760 KB)

## Data Availability

The authors declare that all data supporting the findings of this study are available within the article and its Supplementary Information files or from the corresponding author upon reasonable request.
