# Supplementary material for: Clinical and genetic architecture of a large cohort with auditory neuropathy
Source: Hum Genet. 2024 Mar 8;143(3):293–309. doi: 10.1007/s00439-024-02652-7 (PMC11043192; doi:10.1007/s00439-024-02652-7)
Supplement: Supplementary file 2 — Supplementary file2 (DOCX 3760 KB) [file 439_2024_2652_MOESM2_ESM.docx]

**Supplementary Information**

**Supplementary Fig. 1** Family trees and positive genes identification for familial AN cases

**Supplementary Fig. 2** Audiograms of AN patients from different testing age subgroups

**Supplementary Fig. 3** Correlation of PTA/ASSR and SDS for bilateral AN

**Supplementary Fig. 4** DPOAE of AN patients from different testing age subgroups

**Supplementary Fig. 5** The expression analysis of the AN new gene in human cochlea

**Supplementary Fig. 6** Gene function clustering analysis of the 139 genes

**Supplementary Fig. 7** CAP and SIR score for the patients with or without intervention

**Supplementary Table 1.** Classification rules of patients with positive variants

**Supplementary Table 2.** Supplementary audiological characteristics of bilateral auditory neuropathy cases according to age information

**Supplementary Table 3.** Potential druggability of 23 AN genes with pathogenic or likely pathogenic variants

**Supplementary Table 4.** Novel AN genes

**Supplementary Table 5.** Telephone follow-up of intervention information

**Supplementary Table 6.** Cochlea implant outcome in Genetic Auditory Neuropathy

**
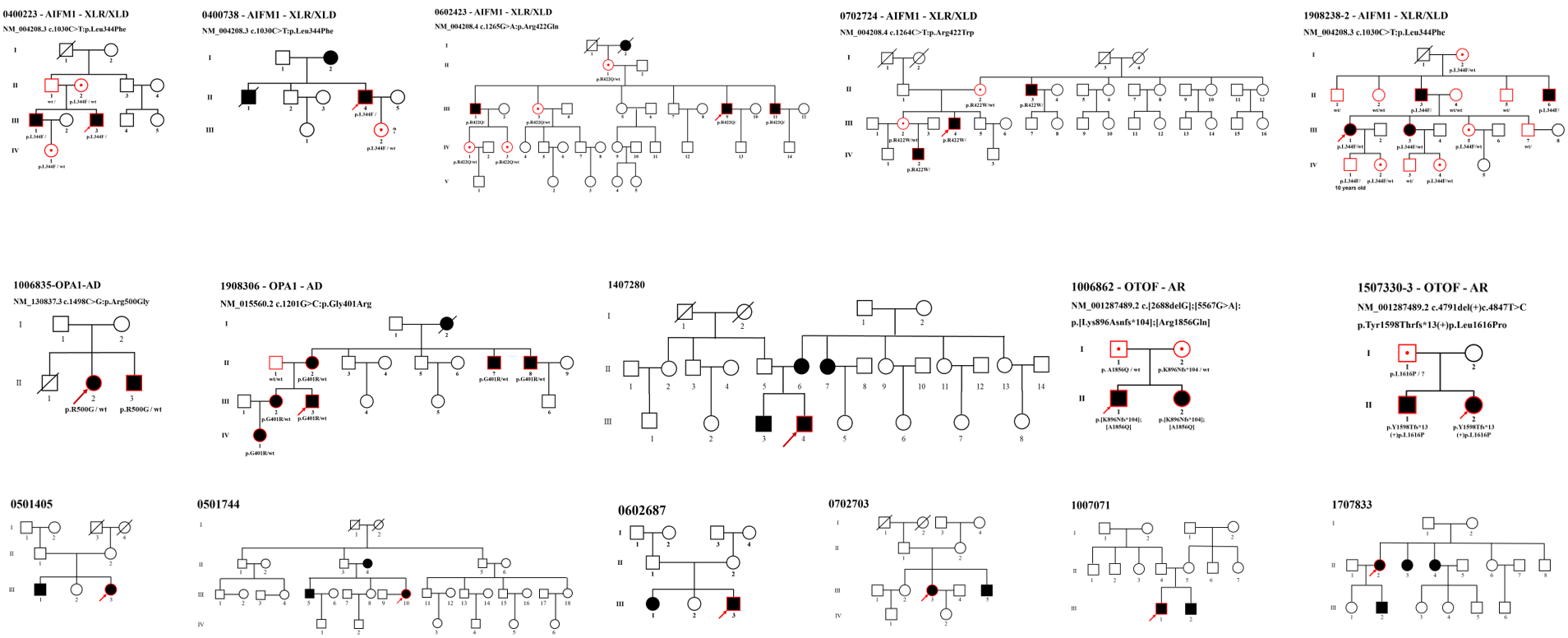
Supplementary Fig. 1** Family trees and positive genes identification for familial AN cases.

**Supplementary Fig. 2** Audiograms of AN patients from different testing age subgroups.

**Supplementary Fig. 3** Correlation of PTA/ASSR and SDS for bilateral AN. L, left; R, right; PTA, pure tone average; ASSR, auditory steady-state response.

**Supplementary Fig. 4** DPOAE of AN patients from different testing age subgroups.

**
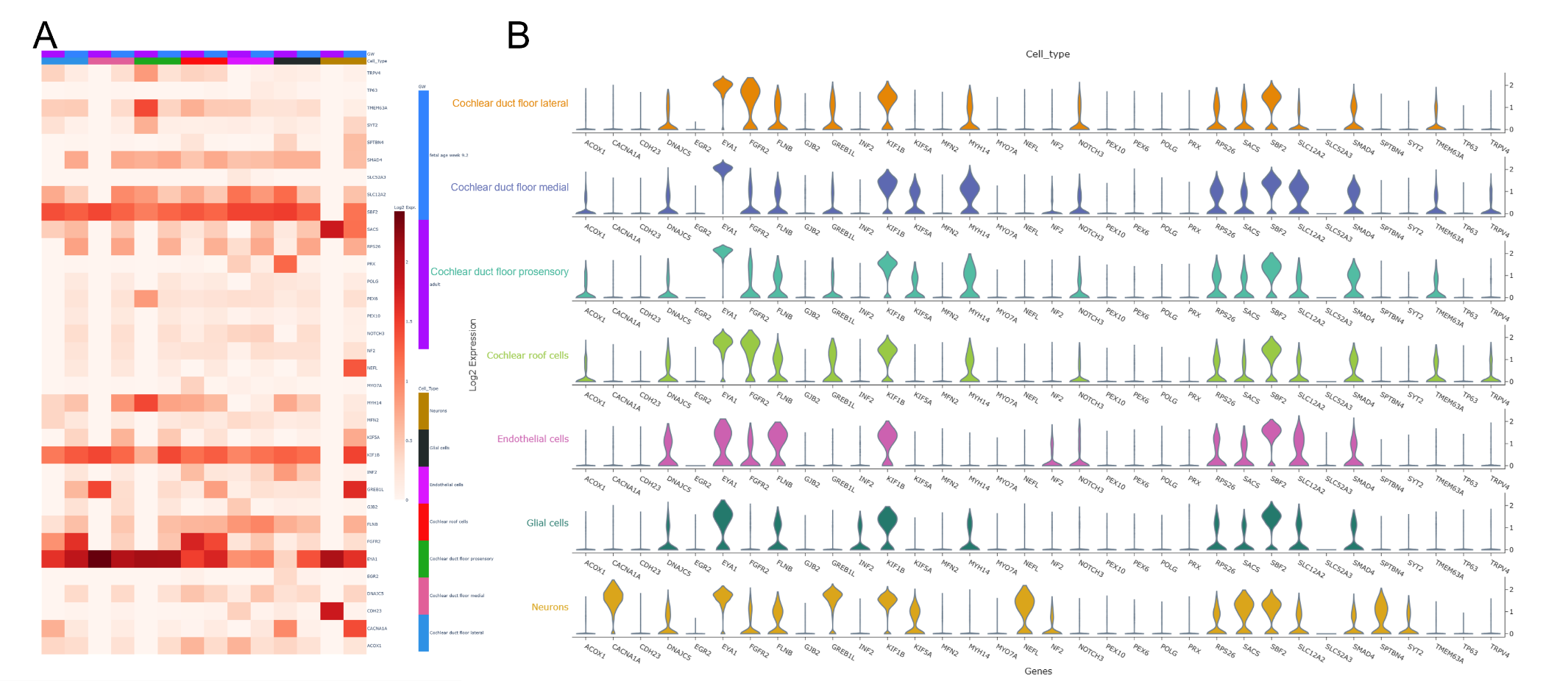
**

**Supplementary Fig. 5** The expression analysis of the AN new gene in human cochlea. **A** Comparative heatmap of the expression between 9.2-week-old infants and adult human cochlea. **B** The expression of the AN new gene in different cells of the adult human cochlea.

**
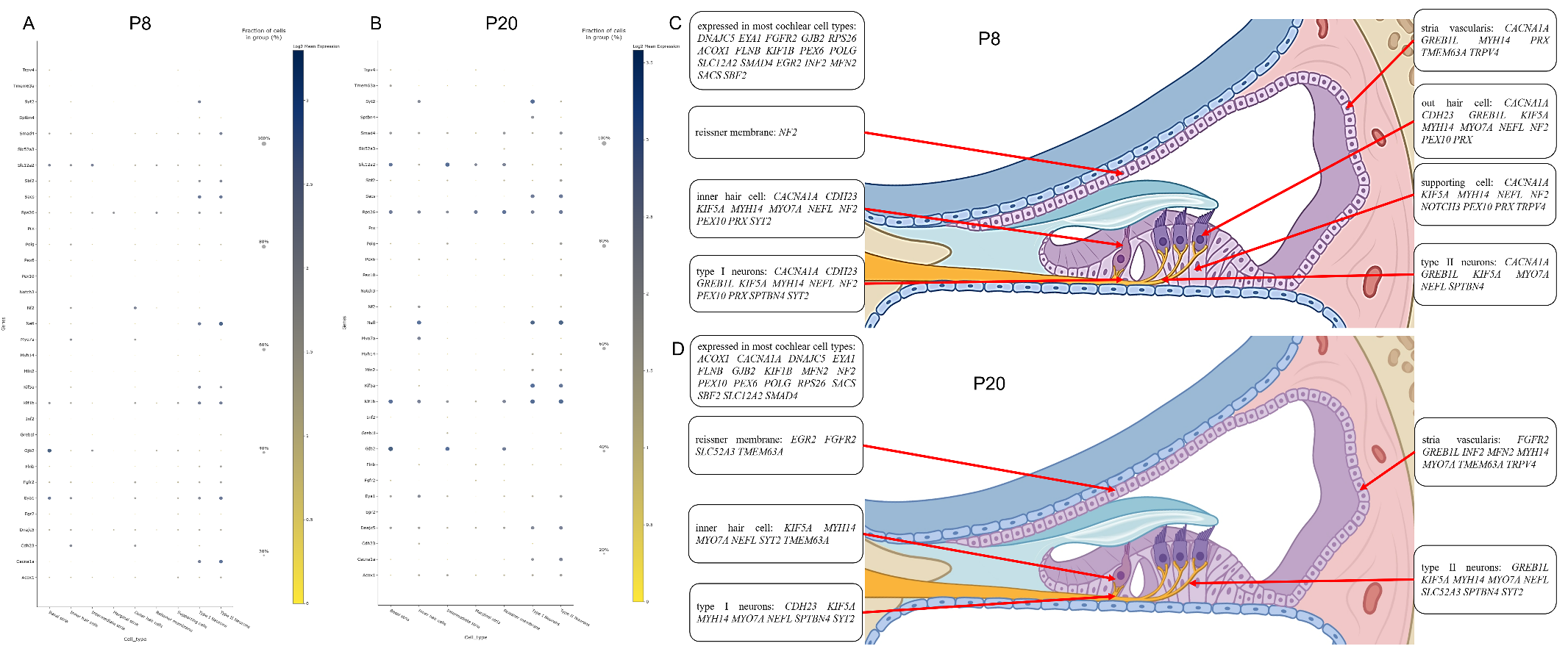
**

**Supplementary Fig. 6** The expression analysis of the AN new gene in mouse cochlea. **A** The expression of different cell types in P8 mice. **B** The expression of different cell types in P20 mice. **C** the gene expression pattern map of the AN new gene in the cochlea in P8 mice. **D** the gene expression pattern map of the AN new gene in the cochlea in P20 mice.

 **Supplementary Fig. 7** CAP and SIR score for the patients with or without intervention. CAP, auditory performance; SIR, speech intelligibility rating; CI, cochlear implantation; HA, hearing aid; NA, patients with no intervention.

**Supplementary Table 1** Classification rules of patients with positive variants

| **Inheritance pattern** | **ACMG classification** | **Heterozygous/Homozygous** | **Combined classification** |
| --- | --- | --- | --- |
| AD | P | Heterozygous | Pathogenic |
| AD | LP | Heterozygous | Likely pathogenic |
| AD | VUS\|P | Heterozygous | Possible pathogenic |
| AD | VUS\|LP | Heterozygous | Possible pathogenic |
| AD | VUS | Heterozygous | unknown |
| AR | P+P | Heterozygous | Pathogenic |
| AR | P+LP | Heterozygous | Pathogenic |
| AR | P+VUS\|P\|LP | Heterozygous | Likely pathogenic |
| AR | P+VUS | Heterozygous | Possible pathogenic |
| AR | LP+LP | Heterozygous | Likely pathogenic |
| AR | LP+VUS\|P\|LP | Heterozygous | Likely pathogenic |
| AR | LP+VUS | Heterozygous | unknown |
| AR | VUS\|P\|LP+VUS | Heterozygous | unknown |
| AR | VUS\|P\|LP+VUS\|P\|LP | Heterozygous | Possible pathogenic |
| AR | VUS+VUS | Heterozygous | unknown |
| AR | P | Homozygous | Pathogenic |
| AR | LP | Homozygous | Likely pathogenic |
| AR | VUS\|P\|LP | Homozygous | Possible pathogenic |
| AR | VUS | * | unknown |
| AR | P | Heterozygous | Carrier |
| AR | LP | Heterozygous | Carrier |
| AR | VUS\|P\|LP | Heterozygous | Carrier |
| XLD | P | Heterozygous | Pathogenic |
| XLD | LP | Heterozygous | Likely pathogenic |
| XLD | VUS\|P\|LP | Heterozygous | Possible pathogenic |
| XLD | VUS | Heterozygous | unknown |
| XLR | P | Heterozygous | Carrier |
| XLR | LP | Heterozygous | Carrier |
| XLR | VUS\|P\|LP | Heterozygous | Carrier |
| XLR | VUS | Homozygous/heterozygous | unknown |
| XLR | P | Homozygous | Pathogenic |
| XLR | LP | Homozygous | Likely pathogenic |
| XLR | VUS\|P\|LP | Homozygous | Possible pathogenic |
| unknown | VUS | * | unknown |
| unknown | * | * | ? |

AD, autosomal dominant; AR, autosomal recessive; XLD, X linked dominant; XLR, X linked recessive.

For genes with both AD and AR inheritance pattern, AD inheritance pattern was first priority.

**Supplementary Table 2** Supplementary audiological characteristics of bilateral auditory neuropathy cases according to age information

|  | Total | ≤3y | >3y | P-value |
| --- | --- | --- | --- | --- |
| **L-PTA severity** |  |  |  | <0.001 |
| Normal | 12 (5.2%) | 0 (0.0%) | 12 (5.9%) |  |
| Mild | 58 (25.0%) | 1 (3.4%) | 57 (28.1%) |  |
| Moderate | 77 (33.2%) | 1 (3.4%) | 76 (37.4%) |  |
| Moderately Severe | 35 (15.1%) | 0 (0.0%) | 35 (17.2%) |  |
| Severe | 17 (7.3%) | 2 (6.9%) | 15 (7.4%) |  |
| Profound | 16 (6.9%) | 12 (41.4%) | 4 (2.0%) |  |
| Total deaf | 17 (7.3%) | 13 (44.8%) | 4 (2.0%) |  |
| **L audiogram** |  |  |  | <0.001 |
| Normal | 2 (0.9%) | 0 (0.0%) | 2 (1.0%) |  |
| Ascending | 122 (52.6%) | 3 (10.3%) | 119 (58.6%) |  |
| Descending | 7 (3.0%) | 2 (6.9%) | 5 (2.5%) |  |
| Flat | 36 (15.5%) | 11 (37.9%) | 25 (12.3%) |  |
| Total deaf | 16 (6.9%) | 12 (41.4%) | 4 (2.0%) |  |
| Tip | 43 (18.5%) | 1 (3.4%) | 42 (20.7%) |  |
| Valley | 6 (2.6%) | 0 (0.0%) | 6 (3.0%) |  |
| **R-PTA severity** |  |  |  | <0.001 |
| Normal | 14 (6.0%) | 1 (3.3%) | 13 (6.4%) |  |
| Mild | 50 (21.5%) | 0 (0.0%) | 50 (24.6%) |  |
| Moderate | 71 (30.5%) | 1 (3.3%) | 70 (34.5%) |  |
| Moderately Severe | 49 (21.0%) | 1 (3.3%) | 48 (23.6%) |  |
| Severe | 16 (6.9%) | 3 (10.0%) | 13 (6.4%) |  |
| Profound | 13 (5.6%) | 9 (30.0%) | 4 (2.0%) |  |
| Total deaf | 20 (8.6%) | 15 (50.0%) | 5 (2.5%) |  |
| **R-Audiogram** |  |  |  | <0.001 |
| Normal | 3 (1.3%) | 1 (3.3%) | 2 (1.0%) |  |
| Ascending | 125 (53.6%) | 3 (10.0%) | 122 (60.1%) |  |
| Descending | 6 (2.6%) | 1 (3.3%) | 5 (2.5%) |  |
| Flat | 29 (12.4%) | 9 (30.0%) | 20 (9.9%) |  |
| Total deaf | 18 (7.7%) | 14 (46.7%) | 4 (2.0%) |  |
| Tip | 43 (18.5%) | 2 (6.7%) | 41 (20.2%) |  |
| Valley | 9 (3.9%) | 0 (0.0%) | 9 (4.4%) |  |
| **Audiogram symmetry** | |  |  | 0.076 |
| N | 44 (19.0%) | 2 (6.9%) | 42 (20.7%) |  |
| Y | 188 (81.0%) | 27 (93.1%) | 161 (79.3%) |  |
| **Presence of ASSR** |  |  |  |  |
| *Left ear* |  |  |  |  |
| 0.5KHz | (107) 81.6 (18.0) 84.0 (74.0-94.0) | (69) 83.8 (15.3) 84.0 (74.0-94.0) | (38) 77.6 (21.8) 79.5 (64.0-94.0) | 0.091 |
| 1KHz* | (107) 86.7 (19.7) 89.0 (79.0-99.0) | (69) 86.6 (19.1) 89.0 (79.0-99.0) | (38) 86.9 (21.0) 89.5 (79.0-99.0) | 0.672 |
| 2KHz | (107) 84.0 (17.7) 87.0 (77.0-97.0) | (69) 80.7 (17.7) 77.0 (67.0-90.0) | (38) 90.1 (16.3) 87.0 (77.0-97.0) | 0.008 |
| 4KHz | (107) 71.1 (15.7) 71.0 (61.0-81.0) | (69) 69.0 (16.3) 70.0 (61.0-75.0) | (38) 74.8 (13.8) 71.0 (71.0-81.0) | 0.067 |
| *Right ear* |  |  |  |  |
| 0.5KHz | (106) 77.7 (15.8) 74.0 (64.0-84.0) | (69) 79.4 (14.0) 77.0 (74.0-90.0) | (37) 74.5 (18.4) 74.0 (64.0-84.0) | 0.131 |
| 1KHz* | (107) 85.0 (18.4) 89.0 (79.0-99.0) | (69) 86.0 (17.3) 89.0 (79.0-99.0) | (38) 83.3 (20.4) 89.0 (79.0-99.0) | 0.67 |
| 2KHz | (106) 80.6 (18.1) 77.0 (67.0-97.0) | (69) 79.3 (17.0) 77.0 (67.0-87.0) | (37) 83.2 (19.9) 87.0 (67.0-97.0) | 0.293 |
| 4KHz | (107) 70.1 (16.3) 71.0 (61.0-81.0) | (69) 67.1 (15.3) 61.0 (61.0-71.0) | (38) 75.5 (16.9) 71.0 (71.0-81.0) | 0.01 |

PTA, pure tone average. ASSR, auditory steady-state response. Variable representation method: (N) Mean (SD) Median (Q1-Q3).

**Supplementary Table 3** Potential druggability of 23 AN genes with pathogenic or likely pathogenic variants

| **Druggable Gene Category** | **Matching Gene Count** | **Matching Gene(s)** | **Non-Matching Gene(s)** |
| --- | --- | --- | --- |
| TRANSPORTER | 9 | *FLNB, GJB2, GJB3, MFN2, PEX10, PEX6, SLC12A2, SLC52A3, WFS1* | *ACOX1, CACNA1A, CDH2, EGR2, EYA1, FGFR2, KIF1B, NF2, NOTCH3, POLG, SACS, SBF2, SMAD4, TMEM63A, TP63, TRPV4* |
| DRUGGABLE GENOME | 8 | *CACNA1A, CDH2, FGFR2, NOTCH3, SACS, SLC12A2, TP63, TRPV4* | *ACOX1, EGR2, EYA1, FLNB, GJB2, GJB3, KIF1B, MFN2, NF2, PEX10, PEX6, POLG, SBF2, SLC52A3, SMAD4, TMEM63A, WFS1* |
| CLINICALLY ACTIONABLE | 6 | *FGFR2, KIF1B, NF2, NOTCH3, SMAD4, TP63* | *ACOX1, CACNA1A, CDH2, EGR2, EYA1, FLNB, GJB2, GJB3, MFN2, PEX10, PEX6, POLG, SACS, SBF2, SLC12A2, SLC52A3, TMEM63A, TRPV4, WFS1* |
| CELL SURFACE | 4 | *CDH2, FGFR2, NOTCH3, TRPV4* | *ACOX1, CACNA1A, EGR2, EYA1, FLNB, GJB2, GJB3, KIF1B, MFN2, NF2, PEX10, PEX6, POLG, SACS, SBF2, SLC12A2, SLC52A3, SMAD4, TMEM63A, TP63, WFS1* |
| ENZYME | 3 | *ACOX1, EYA1, POLG* | *CACNA1A, CDH2, EGR2, FGFR2, FLNB, GJB2, GJB3, KIF1B, MFN2, NF2, NOTCH3, PEX10, PEX6, SACS, SBF2, SLC12A2, SLC52A3, SMAD4, TMEM63A, TP63, TRPV4, WFS1* |
| ION CHANNEL | 3 | *CACNA1A, TMEM63A, TRPV4* | *ACOX1, CDH2, EGR2, EYA1, FGFR2, FLNB, GJB2, GJB3, KIF1B, MFN2, NF2, NOTCH3, PEX10, PEX6, POLG, SACS, SBF2, SLC12A2, SLC52A3, SMAD4, TP63, WFS1* |
| KINASE | 3 | *FGFR2, NF2, SMAD4* | *ACOX1, CACNA1A, CDH2, EGR2, EYA1, FLNB, GJB2, GJB3, KIF1B, MFN2, NOTCH3, PEX10, PEX6, POLG, SACS, SBF2, SLC12A2, SLC52A3, TMEM63A, TP63, TRPV4, WFS1* |
| DRUG RESISTANCE | 2 | *NF2, SMAD4* | *ACOX1, CACNA1A, CDH2, EGR2, EYA1, FGFR2, FLNB, GJB2, GJB3, KIF1B, MFN2, NOTCH3, PEX10, PEX6, POLG, SACS, SBF2, SLC12A2, SLC52A3, TMEM63A, TP63, TRPV4, WFS1* |
| TRANSCRIPTION FACTOR | 2 | *EGR2, TP63* | *ACOX1, CACNA1A, CDH2, EYA1, FGFR2, FLNB, GJB2, GJB3, KIF1B, MFN2, NF2, NOTCH3, PEX10, PEX6, POLG, SACS, SBF2, SLC12A2, SLC52A3, SMAD4, TMEM63A, TRPV4, WFS1* |
| EXTERNAL SIDE OF PLASMA MEMBRANE | 1 | *SLC12A2* | *ACOX1, CACNA1A, CDH2, EGR2, EYA1, FGFR2, FLNB, GJB2, GJB3, KIF1B, MFN2, NF2, NOTCH3, PEX10, PEX6, POLG, SACS, SBF2, SLC52A3, SMAD4, TMEM63A, TP63, TRPV4, WFS1* |

**Supplementary Table 4** Novel AN genes

| **No.** | **GENE** |
| --- | --- |
| 1 | *ACOX1* |
| 2 | *CACNA1A* |
| 3 | *CDH23* |
| 4 | *DNAJC5* |
| 5 | *EGR2* |
| 6 | *EYA1* |
| 7 | *FGFR2* |
| 8 | *FLNB* |
| 9 | *GJB2* |
| 10 | *GREB1L* |
| 11 | *INF2* |
| 12 | *KIF1B* |
| 13 | *KIF5A* |
| 14 | *MFN2* |
| 15 | *MYH14* |
| 16 | *MYO7A* |
| 17 | *NEFL* |
| 18 | *NF2* |
| 19 | *NOTCH3* |
| 20 | *PEX10* |
| 21 | *PEX6* |
| 22 | *POLG* |
| 23 | *PRX* |
| 24 | *RPS26* |
| 25 | *SACS* |
| 26 | *SBF2* |
| 27 | *SLC12A2* |
| 28 | *SLC52A3* |
| 29 | *SMAD4* |
| 30 | *SPTBN4* |
| 31 | *SYT2* |
| 32 | *TMEM63A* |
| 33 | *TP63* |
| 34 | *TRPV4* |

**Supplementary Table 5** Telephone follow-up of intervention information

**Telephone follow-up of intervention information**

1. Medical record No：

2. Name：

3. Gender：

4. Birth date：

5. Age of onset：

6. Age of follow-up：

7. Phone No：

Recorder： Recording time：

Recording unit：

| Questions | Response (Please check the "□" ) | Note |
| --- | --- | --- |
| 1. Intervention | 1. No Intervention □ 2. Hearing aid (HA) □ 3. Cochlear implant (CI) □ 4. Dual-mode □ |  |
| 1. Side of HA | 1. Left ear □ Time/Age of onset： 2. Right ear □ Time/Age of onset： 3. Bilateral ear □ Time/Age of onset： |  |
| 1. Side of CI | 1. Left ear □ Time/Age of onset： 2. Right ear □ Time/Age of onset： 3. Bilateral ear □ Time/Age of onset： |  |
| 1. Categories of auditory performance   (CAP) | 1. No awareness of environmental sounds (0) □ 2. Awareness of environmental sounds (1) □ 3. Response to speech sounds (2) □ 4. Identification of environmental sounds (3) □ 5. Discrimination of speech sounds without lip reading (4) □ 6. Understanding of common phrases without lip reading (5) □ 7. Understanding of conversation without lip reading (6) □ 8. Use of telephone with known speaker (7) □ |  |
| 1. Speech Intelligibility Ratings   (SIR) | 1. Connected speech is unintelligible. Prerecognizable words in spoken language, the primary mode of communication may be manual (1) □ 2. Connected speech is unintelligible. Intelligible speech is developing in single words when context and lipreading cues are available (2) □ 3. Connected speech is intelligible to a listener who concentrates and lipreads (3) □ 4. Connected speech is intelligible to a listener who has a little experience of a deaf person’s speech (4) □ 5. Connected speech is intelligible to all listeners. The child is understood easily in everyday contexts (5) □ |  |
| 6. Intention to review | 1. Yes □ Specific/Approximate time： 2. Uncertainty □ 3. No □ |  |
| 7. Loss to follow-up | 1. Lost call □ 2. [Wrong number](C:/Users/Up/AppData/Local/youdao/dict/Application/9.1.2.0/resultui/html/index.html#/javascript:;) □ 3. Refusal to cooperate □ |  |

**电话随访信息采集表 —— 干预情况**

1、病历号：2、姓名：3、性别：4、出生日期：

5、发病年龄：6、随访年龄：7、信息记录者：

| 项目 | 请在“□”处打勾 | 备注 |
| --- | --- | --- |
| 1、干预情况 | 1、未干预 □  2、助听器 □  3、人工耳蜗 □  4、双模式 □ |  |
| 2、助听器侧别 | 1. 左侧 □ 开始时间或年龄： 2. 右侧 □ 开始时间或年龄： 3. 双侧 □ 开始时间或年龄： |  |
| 3、人工耳蜗植入侧别 | 1. 左侧 □ 开始时间或年龄： 2. 右侧 □ 开始时间或年龄： 3. 双侧 □ 开始时间或年龄： |  |
| 4、听觉能力分级  （CAP） | 1、不能察觉到环境声（0级） □  2、能察觉到环境声（1级） □  3、对言语声有反应（2级） □  4、能识别出环境声（3级） □  5、不借助唇读能分辨一些言语声（4级）□  6、不借助唇读能理解常用短语（5级） □  7、不借助唇读能理解交谈内容（6级） □  8、能和认识的人打电话（7级） □ |  |
| 5、言语可懂度分级  （SIR） | 1、其连贯的言语（短语或句子）不能被听懂。其口语中的词汇不易被识别，日常交流的主要方式为手势（1级） □  2、其连贯的言语不能被听懂。当结合上下文和唇读线索时，其言语的单个词汇可逐渐被听懂（2级） □  3、当集中注意力并结合唇读线索时，其连贯的言语能被听懂（3级）□  4、其连贯的言语能被不熟悉的人听懂（4级） □  5、其连贯的言语能被所有人听懂。日常语境下，儿童的言语能被轻易听懂（5级） □ |  |
| 6、复查意向 | 1、有意向 □具体日期（或大概时间段）：  2、暂无法确定 □  3、明确无意向 □ |  |
| 7、失访 | 1、多次尝试未接通 □  2、号码错误 □  3、拒绝配合 □ |  |

信息记录时间：

信息记录单位：

联系电话：

**Supplementary Table 6** Cochlea implant outcome in Genetic Auditory Neuropathy

| **Site of lesion** | **Locus** | **Gene** | **Phenotype** | **ClinGen** | **Previous studies** | | **This study** | | | |  |
| --- | --- | --- | --- | --- | --- | --- | --- | --- | --- | --- | --- |
|  |  |  |  |  | CI cases | CI effect | Cases | CI Cases | CAP | SIR | Ref |
| Pre-synaptic | Cochlea IHC | *SLC17A8* | ADNSHL | Strong | 1 | significant | 0 | 0 |  |  | (Wu et al. 2011) |
|  | IHC ribbon synapse | *OTOF* | AN/ARNSHL | Definitive | 61 | significant | 29 | 15 | 5.93±0.85  6(1) | 3.40±1.36  3(1.5) | (Miyagawa et al. 2016; Rodríguez-Ballesteros et al. 2008; Rouillon et al. 2006; Wu et al. 2011; Zheng and Liu 2020) |
|  |  | *CACNA1D* | SANND syndrome | Moderate | NA | significant | 3 | 0 |  |  |  |
| Post-synaptic | Dendrite | *OPA1* | DOA, or DOA and AN (DOA+) | NA | 7 | significant | 10 | 0 |  |  | (Chaudhry et al. 2020; Huang et al. 2009; Rance and Barker 2008; Starr et al. 2004) |
|  |  | *DIAPH3* | ADNSHL | Limited | 3 | significant | 0 | 0 |  |  | (Shearer and Hansen 2019) |
|  |  | *ATP1A3* | AN/CAPOS syndrome | NA | 2 | significant | 7 | 0 |  |  | (Han et al. 2017) |
|  |  | *ROR1* | ARNSHL, inner ear malformation | Limited | 1 | significant | 0 | 0 |  |  | (Diaz-Horta et al. 2016) |
|  | Ganglion cells and proximal axon | *TIMM8A* | DDON syndrome | Definitive | 2 | Poor | 1 | 0 |  |  | (Brookes et al. 2008) |
|  |  | *PJVK* | ARNSHL | Definitive | 3 | Poor | 0 | 0 |  |  | (Wu et al. 2015) |
|  |  | *AIFM1* | X-linked AN; Cowchock syndrome | Definitive | - | Poor | 32 | 1 | 5 | 5 | (Zong et al. 2015) |
|  |  | *NARS2* | ARNSHL; Leigh syndrome (progressive neurodegenerative disease) | Limited | - | Poor | 1 | 0 |  |  | (Shearer and Hansen 2019) |
|  |  | *MPZ, PMP22* | CMT; hereditary sensory and motor neuropathy | NA | - | Poor | 0  2 | 0  0 |  |  | (Cif et al. 2013) |

CMT, Charcot–Marie–Tooth

Brookes J, Kanis A, Tan L, Tranebjaerg L, Vore A, Smith R (2008) Cochlear implantation in deafness-dystonia-optic neuronopathy (DDON) syndrome. International journal of pediatric otorhinolaryngology 72: 121-6. https://doi.org/10.1016/j.ijporl.2007.08.019

Chaudhry D, Chaudhry A, Muzaffar J, Monksfield P, Bance M (2020) Cochlear Implantation Outcomes in Post Synaptic Auditory Neuropathies: A Systematic Review and Narrative Synthesis. The journal of international advanced otology 16: 411-431. https://doi.org/10.5152/iao.2020.9035

Cif L, Gonzalez V, Garcia-Ptacek S, James S, Boetto J, Seychelles A, Roujeau T, Moura De Ribeiro A, Sillon M, Mondain M, Coubes P (2013) Progressive dystonia in Mohr-Tranebjaerg syndrome with cochlear implant and deep brain stimulation. Movement disorders : official journal of the Movement Disorder Society 28: 737-8. https://doi.org/10.1002/mds.25519

Diaz-Horta O, Abad C, Sennaroglu L, Foster J, DeSmidt A, Bademci G, Tokgoz-Yilmaz S, Duman D, Cengiz F, Grati M, Fitoz S, Liu X, Farooq A, Imtiaz F, Currall B, Morton C, Nishita M, Minami Y, Lu Z, Walz K, Tekin M (2016) ROR1 is essential for proper innervation of auditory hair cells and hearing in humans and mice. Proceedings of the National Academy of Sciences of the United States of America 113: 5993-8. https://doi.org/10.1073/pnas.1522512113

Han K, Oh D, Lee S, Lee C, Han J, Kim M, Park H, Park M, Kim N, Lee J, Yi E, Kim J, Kim J, Chae J, Oh S, Park W, Choi B (2017) ATP1A3 mutations can cause progressive auditory neuropathy: a new gene of auditory synaptopathy. Scientific reports 7: 16504. https://doi.org/10.1038/s41598-017-16676-9

Huang T, Santarelli R, Starr A (2009) Mutation of OPA1 gene causes deafness by affecting function of auditory nerve terminals. Brain research 1300: 97-104. https://doi.org/10.1016/j.brainres.2009.08.083

Miyagawa M, Nishio S, Usami S (2016) A Comprehensive Study on the Etiology of Patients Receiving Cochlear Implantation With Special Emphasis on Genetic Epidemiology. Otology & neurotology : official publication of the American Otological Society, American Neurotology Society [and] European Academy of Otology and Neurotology 37: e126-34. https://doi.org/10.1097/mao.0000000000000936

Rance G, Barker E (2008) Speech perception in children with auditory neuropathy/dyssynchrony managed with either hearing AIDS or cochlear implants. Otology & neurotology : official publication of the American Otological Society, American Neurotology Society [and] European Academy of Otology and Neurotology 29: 179-82. https://doi.org/10.1097/mao.0b013e31815e92fd

Rodríguez-Ballesteros M, Reynoso R, Olarte M, Villamar M, Morera C, Santarelli R, Arslan E, Medá C, Curet C, Völter C, Sainz-Quevedo M, Castorina P, Ambrosetti U, Berrettini S, Frei K, Tedín S, Smith J, Cruz Tapia M, Cavallé L, Gelvez N, Primignani P, Gómez-Rosas E, Martín M, Moreno-Pelayo M, Tamayo M, Moreno-Barral J, Moreno F, del Castillo I (2008) A multicenter study on the prevalence and spectrum of mutations in the otoferlin gene (OTOF) in subjects with nonsyndromic hearing impairment and auditory neuropathy. Human mutation 29: 823-31. https://doi.org/10.1002/humu.20708

Rouillon I, Marcolla A, Roux I, Marlin S, Feldmann D, Couderc R, Jonard L, Petit C, Denoyelle F, Garabédian E, Loundon N (2006) Results of cochlear implantation in two children with mutations in the OTOF gene. International journal of pediatric otorhinolaryngology 70: 689-96. https://doi.org/10.1016/j.ijporl.2005.09.006

Shearer A, Hansen M (2019) Auditory synaptopathy, auditory neuropathy, and cochlear implantation. Laryngoscope investigative otolaryngology 4: 429-440. https://doi.org/10.1002/lio2.288

Starr A, Isaacson B, Michalewski H, Zeng F, Kong Y, Beale P, Paulson G, Keats B, Lesperance M (2004) A dominantly inherited progressive deafness affecting distal auditory nerve and hair cells. Journal of the Association for Research in Otolaryngology : JARO 5: 411-26. https://doi.org/10.1007/s10162-004-5014-5

Wu C, Lin Y, Liu T, Lin K, Yang W, Hsu C, Chen P, Wu C (2015) Identifying Children With Poor Cochlear Implantation Outcomes Using Massively Parallel Sequencing. Medicine 94: e1073. https://doi.org/10.1097/md.0000000000001073

Wu C, Liu T, Wang S, Hsu C, Wu C (2011) Genetic characteristics in children with cochlear implants and the corresponding auditory performance. The Laryngoscope 121: 1287-93. https://doi.org/10.1002/lary.21751

Zheng D, Liu X (2020) Cochlear Implantation Outcomes in Patients With OTOF Mutations. Front Neurosci 14: 447. https://doi.org/10.3389/fnins.2020.00447

Zong L, Guan J, Ealy M, Zhang Q, Wang D, Wang H, Zhao Y, Shen Z, Campbell C, Wang F, Yang J, Sun W, Lan L, Ding D, Xie L, Qi Y, Lou X, Huang X, Shi Q, Chang S, Xiong W, Yin Z, Yu N, Zhao H, Wang J, Wang J, Salvi R, Petit C, Smith R, Wang Q (2015) Mutations in apoptosis-inducing factor cause X-linked recessive auditory neuropathy spectrum disorder. Journal of medical genetics 52: 523-31. https://doi.org/10.1136/jmedgenet-2014-102961
